# Engineering Oxygen‐Terminated Hexagonal MBene for Accelerated Lithium Migration and Exceptional Lithium‐Ion Battery Performance

**DOI:** 10.1002/advs.202513674

**Published:** 2025-09-26

**Authors:** Qing Shen, LeYang Wang, Pengjie Jiang, Jinming Wang, Yaxin Di, Hui Mei, Junjie Wang, Laifei Cheng

**Affiliations:** ^1^ State Key Laboratory of Solidification Processing School of Materials Science and Engineering Northwestern Polytechnical University Xi'an Shaanxi 710072 P. R. China; ^2^ Science and technology on Thermostructural Composite Materials Laboratory School of Materials Science and Engineering Northwestern Polytechnical University Xi'an Shaanxi 710072 P. R. China; ^3^ School of Materials Science and Engineering Northwestern Polytechnical University Xi'an Shaanxi 710072 P. R. China; ^4^ State Key Laboratory of Chem/Biosensing and Chemometrics College of Chemistry and Chemical Engineering Hunan University Changsha 410082 P. R. China

**Keywords:** high‐rate performance, LIBs, molten salt etching, TiBT_x_ h‐MBene, Zn‐In intermediate phase

## Abstract

Ultrathin 2D hexagonal transition metal borides (*h*‐MBenes) hold significant promise for advancing energy storage technologies. Herein, with cost‐effective methods and earth‐abundant metals, the experimental feasibility of atomically thin Ti‐based 2D *h*‐MBenes (TiBT_x_) for lithium‐ion battery application is reported for the first time. These thin‐layer nanosheets are synthesized by using ZnCl_2_ molten salt as Ti_2_InB_2_ etchant, followed with a delaminated intercalation of tetrabutylammonium hydroxide (denoted as d‐TiBT_x_). The formation of disorderly low‐boiling‐point Zn‐In intermediate phase is revealed to significantly reduce the In migration energy barrier and accelerate the lattice‐In release from parent Ti_2_InB_2_ under low‐temperature. Moreover, in‐depth analyses reveal that the formation of O‐termination on the atomic d‐TiBT_x_ surface endows d‐TiBT_x_ *h*‐MBene with exceptional lithium‐ion migration, achieving an impressive specific capacity of 530 mAh g^−1^ at 0.1 A g^−1^ and an exceptional rate capability of 120 mAh g^−1^ at 10 A g^−1^. Notably, a lithium full‐cell paired with a LiFePO_4_ cathode achieves an impressive energy density of 425 Wh kg^−1^ and retains 94.3% of its capacity after 100 cycles, sufficient to power a toy car under normal operation. This work confirms the usefulness of ultrathin Ti‐based 2D MBenes, paving the way for innovatively harnessing the potential application of *h*‐MBenes.

## Graphical abstract

The synthesis of ultrathin Ti‐based 2D hexagonal transition metal borides (TiBT_x_ *h*‐MBene) via ZnCl_2_ molten salt and the formed disorderly low‐boiling‐point Zn‐In intermediate, followed by TBAOH delamination. The delaminated structure, with O‐ and Cl‐terminations, improves Li‐ion transport, achieving 530 mAh g^−1^ at 0.1 A g^−1^ and 120 mAh g^−1^ at 10 A g^−1^, with excellent cycling stability in lithium‐ion batteries.

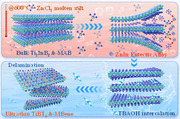

## 1 Introduction

As a flourishing branch of 2D nanomaterials, ultrathin 2D transition metal borides (MBenes) have garnered significant attention in energy conversion and storage, due to their large specific surface area, abundant reactive sites, good electrical conductivity, and high mechanical strength.^[^ ^1^, ^2^ ^]^ MBenes are commonly obtained by selectively etching the A atomic layer in the parent MAB (M: transition metal, A: A group element, B:boron) phase.^[^ ^3^, ^4^ ^]^ However, practical fabrication of atomically thick MBenes have been constrained by the difficulty in identifying suitable parent MAB phases that can be selectively etched to remove A layers for exfoliation. Several attempts have been made to synthesize 2D orthorhombic MBenes (*ort*‐MBenes) from corresponding ternary orthorhombic MAB (*ort*‐MAB) phases,^[^ ^5^, ^6^, ^7^, ^8^, ^9^, ^10^ ^]^ but the limited anisotropy in chemical bonding within *ort*‐MAB phases complicates the selective etching process necessary for preparing nanosheet‐like *ort*‐MBenes.

Taking for the availability of hexagonally MAB phases (*h*‐MAB),^[^ ^11^, ^12^ ^]^ the A layers in *h*‐MAB phases exhibit notable chemical bonding anisotropy compared to *ort*‐MAB, making the preparation of sub‐nanometer 2D MBenes accessible on a laboratory scale.^[^ ^10^ ^]^ Currently, 133 ternary *h*‐MAB phases have been predicted to be synthesizable, with 81 expected to exfoliate into 20 stable 2D hexagonal MBenes (*h*‐MBenes).^[^ ^11^, ^13^ ^]^ Notably, the Lewis Acid Molten Salt (LAMS) strategy, pioneered by Prof. Huang's group, has established a robust foundation for the synthesis of MBenes.^[^ ^14^ ^]^ This method relies on a redox‐coupled reaction between the A‐site element of a MAX (M: transition metal, A: A group element, X: C or N) phase precursor and the cation in the Lewis acidic molten salt. By carefully tuning the composition of the MAX precursor and the Lewis acid melt, selective etching of the A‐site element can be achieved, leading to the formation of 2D MXenes. More recently, this strategy has been extended to the synthesis of *h*‐MBenes. For example, the first *h*‐MBene, HfBO, was successfully synthesized by etching the ternary *h*‐MAB phase Hf_2_InB_2_ using CuCl_2_ molten salt,^[^ ^13^ ^]^ demonstrating the feasibility of LAMS for processing *h*‐MAB phases. Furthermore, quaternary *h*‐MAB phases such as (Mo_2/3_Y_1/3_)_2_AlB_2_ have emerged as promising precursors for 2D *h*‐MBene synthesis.^[^ ^15^ ^]^ In a subsequent study, Prof. Huang's group employed CuBr_2_ molten salt to selectively remove both Al and Y atoms from (Mo_2/3_Y_1/3_)_2_AlB_2_, yielding layered, conductive *h*‐MBenes with ordered metal vacancies and Br‐terminated surfaces.^[^ ^14^ ^]^ Both HfBO and Mo_4/3_B_2_T_x_ MBenes exhibit great potential as electrode materials and electrocatalysts because of 2D structure.^[^ ^13^, ^16^ ^]^ Despite this progress, experimental methods for etching *h*‐MAB phases to obtain the ultrathin *h*‐MBenes, remain in the nascent stages for energy‐storge materials. In addition, a deep insight into the etching mechanism is highly desired, which is of great significance for ultrathin 2D MBenes development.

Moreover, a crucial factor in the economic feasibility of 2D MBene materials is the constituent element abundance in the Earth's crust. Titanium of the Earth's crust is approximately quadruple that Hf and Mo.^[^ ^17^, ^18^ ^]^ Therefore, focusing on Ti‐based *h*‐MBenes could significantly accelerate the development of the 2D MBene material family. The first discovered *h*‐MAB phase, Ti_2_InB_2_, holds promise for producing 2D TiB *h*‐MBenes through selective removal of the indium (In) layers. Experimental work has successfully produced layered boride TiB by dealloying Ti_2_InB_2_ at high temperatures (1050 °C) under vacuum.^[^ ^12^ ^]^ Unfortunately, this process induces a phase transition from hexagonal to orthorhombic symmetry in TiB. Therefore, exploring innovative etching methods is still necessary to obtain the high‐purity Ti‐based 2D *h*‐MBenes for large‐scale practical applications.

**Figure 1:**
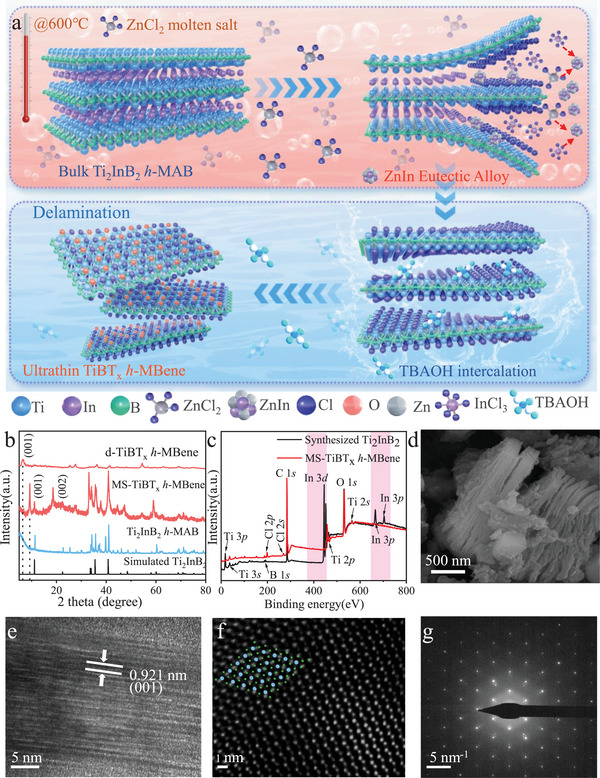
Characterization of pristine Ti_2_InB_2_ and TiBT_x_ *h*‐MBenes. a) Schematic illustration of the preparation of 2D delaminated TiBT_x_ *h*‐MBen; b) XRD of Ti_2_InB_2_, MS‐TiBT_x_, and d‐TiBT_x_ nanosheets; c) XPS survey spectrum of Ti_2_InB_2_ and MS‐TiBT_x_; d) SEM, e) HRTEM, f) HAADF, g) SAED images of MS‐TiBT_x_.

Inspired by these considerations, a significant breakthrough was achieved with the development of energy‐efficient methods for synthesizing ultrathin Ti‐based 2D *h*‐MBene, specifically tailored for use as anode materials in non‐aqueous Li‐ion batteries. Utilizing ZnCl_2_ molten salt as an etchant enables the penetration of low‐melting‐point Cl^−^ and Zn^2+^ penetration into the In atomic layer of Ti_2_InB_2_, leading to the formation of a disordered In‐Zn alloy structure due to the strong chemical reactivity between InCl_3_ and ZnCl_2_. The formation of the In‐Zn intermediate phase significantly lowers the migration energy barrier for In atoms, thereby facilitating the release of lattice In atoms from the parent *h*‐MAB under low‐temperature annealing conditions (**Figure** **1**a). Through delaminated intercalation using tetrabutylammonium hydroxide (TBAOH), double‐layered d‐TiBT_x_ (1.3 nm) was successfully synthesized. Detailed analysis revealed that the d‐TiBT_x_ *h*‐MBene, featuring O‐ and Cl‐termination species introduced during the etching and delamination process, exhibited significantly enhanced electrochemical performance, particularly a high Li^+^ storage capacity in non‐aqueous electrolytes. This work addresses a critical challenge in MBene research—developing ultrathin MBenes from Earth‐abundant elements—and establishes a solid foundation for practical applications as high‐energy‐density electrode materials in lithium batteries.

## 2 Results and Discussion

### 2.1 Synthesis of MS‐TiBT_x_ *h*‐MBene

The etching process began with heating a mixture of Ti_2_InB_2_ powders and ZnCl_2_ in a 1:10 molar ratio within a vacuum atmosphere inside a tube furnace. At 600 °C, the Ti_2_InB_2_ *h*‐MAB precursor was immersed in molten ZnCl_2_, which has a melting point of 280 °C. The Zn^2+^ ions as a Lewis acid, preferentially remove In layers from Ti_2_InB_2_ due to the weaker Ti─In bonds rather than the strong Ti─B bonds. X‐ray diffraction (XRD) confirms that the prepared Ti_2_InB_2_ matches well with prior studies,^[^ ^11^ ^]^ indicating the *h*‐MAB Ti_2_InB_2_ phase synthesis with only minor byproducts successfully (Figure S1, Supporting Information). Furthermore, the impurity phases (Ti_3_In_4_, Ti_3_In, and TiB_2_) within Ti_2_InB_2_ were almost entirely dissolved in the molten ZnCl_2_ (Figure S2, Supporting Information), showing the emanation of high‐purity etched products (denoted as TiBT_x_, T_x_ being ‐Cl or ‐O) with minimal oxidative byproducts after 8 h etching. Subsequently, the etched materials, with exposed layers, are intercalated into TBAOH for 24 h for a further delamination process to obtain the atomically thin *h*‐MBene TiBT_x_ nanosheets (referred as d‐TiBT_x_), following with 6 h sonication under argon atmosphere. Detailed synthesis procedures are outlined in the Experimental Section.

Figure 1b displays that, comparing the XRD patterns of Ti_2_InB_2_, the shift of (001) facet diffraction in the MS‐TiBT_x_ *h*‐MBene from 2*θ* = 11.19° to 9.36° indicates an increase in the d‐spacing from 7.91 to 9.44 Å. This shift reflects the successful expansion of the interlayer distance, characteristic of MXene‐like 2D materials, achieved through Lewis acidic molten salt etching. Notably, the (00l) peak of MS‐TiBT_x_ *h*‐MBene at 2*θ* = 9.36° is sharp and intense, suggesting that the sheets are well‐aligned after etching, in contrast to the broader peaks typically observed in previous studies. Following TBAOH treatment and freeze‐drying, the (001) peak of d‐TiBT_x_ *h*‐MBene shifts further to a lower angle of 2*θ* = 6.91°, corresponding to 12.82 Å d‐spacing. Despite this shift, the XRD pattern of d‐TiBT_x_ *h*‐MBene retains the main peaks of MS‐TiBT_x_ *h*‐MBene, confirming the structural integrity TiBT_x_. The reduced intensity of the (001) and (002) peaks in d‐TiBT_x_ *h*‐MBene can be ascribable to a changeable orientation of ultrathin nanosheets with the TBAOH‐treated and freeze‐dried flakes.^[^ ^19^ ^]^

The XPS survey spectra of the Ti_2_InB_2_ and MS‐TiBT_x_ *h*‐MBene were analyzed to investigate changes in chemical composition during the etching process (Tables S1, S2, Supporting Information; Figure 1c). As shown in Figure 1c, the In signal absence in the XPS spectrum of MS‐TiBT_x_ confirms the successful removal of In atom through the Lewis acid etching reaction. Additionally, new signals observed at 270 and 200 eV are attributed to Cl 2*s* and Cl 2*p*, respectively, while the signal at 530 eV corresponds to O 1*s*.^[^ ^20^ ^]^ These observations indicate the emergence of O‐ and Cl‐terminations on the surface of the prepared MS‐TiBT_x_. The XPS results reveal that the atomic percentages of Cl and O in the MS‐TiBT_x_ are ≈5.68% and 19.59% (Table S2, Supporting Information), respectively. Also, the XPS analysis shows an atomic ratio of Ti:In of 1:0.075 in MS‐TiBT_x_ *h*‐MBene, indicating the successful transformation from Ti_2_InB_2_ to MS‐TiBT_x_ *h*‐MBene. This result is corroborated by Inductively Coupled Plasma Optical Emission Spectroscopy (ICP‐OES, Table S2, Supporting Information), which reveals an 1:0.037 atomic ratio of Ti:In in the same sample. These findings suggest that ≈85% (according to XPS) or 92.6% (according to ICP‐OES) of the Ti_2_InB_2_ was converted into *h*‐MBene. Additionally, XPS and ICP‐OES analyses determined the Ti:B ratio in MS‐TiBT_x_ *h*‐MBene to be 1:1.084 and 1:0.899, respectively, and In element is negligible. These analyses indicate that the lattice In atom from the parent *h*‐MAB is exfoliated successfully.

The morphology of Ti_2_InB_2_ and MS‐TiBT_x_ *h*‐MBene is examined using Scanning Electron Microscopy (SEM) and Transmission Electron Microscopy (TEM). Ti_2_InB_2_ exhibits a typical densely stacked structure similar to other MAX/*h*‐MAB phases (Figure S4, Supporting Information). After treatment with ZnCl_2_, MS‐TiBT_x_ *h*‐MBene reveals a layered structure with flakes arranged in an array (Figure 1d). The small particles observed on the surface of *h*‐MBene are likely oxidative byproducts or residues from the synthesis and etching processes. This expanded structure results in a significantly larger specific surface area for MS‐TiBT_x_ *h*‐MBene (28 m^2^ g^−1^) compared to the pristine Ti_2_InB_2_ (3.8 m^2^ g^−1^, Figure S5, Supporting Information). Elemental mapping obtained from Energy‐dispersive X‐ray spectroscopy (EDS, Figure S6, Supporting Information) shows that Ti, In, and B are uniformly distributed in the Ti_2_InB_2_ precursor, while Ti, B, Cl, and O are uniformly distributed in MS‐TiBT_x_ *h*‐MBene. Comparison of EDS results (Figure S7, Supporting Information) between the Ti_2_InB_2_ precursor and MS‐TiBT_x_ confirms the successful removal of In atoms, as indicated by the significant reduction of In signal in MS‐TiBT_x_ *h*‐MBene.

High‐Resolution Transmission Electron Microscopy (HRTEM) reveals that the interlayer spacing in MS‐TiBT_x_ *h*‐MBene increases to 9.21 Å (Figure 1e), which is consistent with the XRD result of 9.44 Å after the removal of In layer atoms by ZnCl_2_ etching (Figure 1b). The High‐Angle Annular Dark‐Field Scanning Transmission Electron Microscopy (HAADF‐STEM) image shows a close‐packed hexagonal structure with varying atomic contrasts (Figure 1f), where three stronger contrasts likely correspond to Ti atoms and the weaker contrasts to Cl atoms, aligning with the hexagonal simulated structure of TiBT_x_ (inserted in Figure 1f). The Selected Area Electron Diffraction (SAED) pattern (Figure 1g) exhibits distinct reflections corresponding to a hexagonal crystal structure, demonstrating that the ZnCl_2_ etching treatment did not affect the crystallinity of MS‐TiBT_x_ *h*‐MBene. Since EDS is limited for detecting low‐Z elements such as boron,^[^ ^16^ ^]^ Electron Energy Loss Spectroscopy (EELS) is employed for further verification. As seen, EELS point scans from different regions of MS‐TiBT_x_ reveal a boron K edge at ≈187 eV, confirming the presence of boron (Figure S8, Supporting Information), and Ti:B molar ratio measured by EELS is found to be 1:0.968, which is very close to the Ti:B ratio of 1:1 in Ti_2_InB_2_.

Combining measurements from XPS (1:0.899), ICP‐OES (1:1.084), and EELS (1:0.968), Ti:B ratio of MS‐TiBT_x_ flakes is determined to be ≈1:1 (Table S2, Supporting Information). These results collectively confirm that the In layer of Ti_2_InB_2_ *h*‐MAB phase is removed along with Cl‐ and O‐termination incorporation. Also, structural and morphological analyses using XRD, SEM, and HAADF‐STEM demonstrate that a layered hexagonal structure was achieved following the selective removal of In from Ti_2_InB_2_. These comprehensive observations indicate the successful synthesis of high‐purity hexagonal TiBT_x_ MBene (*h*‐MBene) with Cl and O surface groups, Ti_1.22_B_1.08_Cl_1.02_O_0.49_ (Table S3, Supporting Information).

### 2.2 Overview on the Ti_2_InB_2_ Etching Mechanism

**Figure 2:**
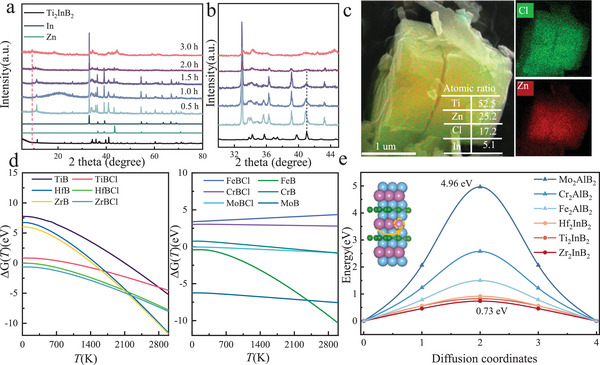
Etching mechanism of *h*‐MAB Ti_2_InB_2_. a) XRD patterns of Ti_2_InB_2_/ZnCl_2_ with the b) magnified diffraction at 2*θ* = 30–45°; c) EDS mapping analysis for the 0.5 h sample; d) Gibbs free energies of formation (ΔG(T)) for MBenes (metal borides) and Cl‐MBenes across the temperature range 0 K‐3000 K; e) Diffusion energy barriers for A atoms (In or Al) migration within the A layers of *h*‐MAB and *ort*‐MAB phases.

For an exploration of the etching mechanism of Ti_2_InB_2_, both density functional theory (DFT) calculations and experiments were conducted. Compared to other representative chloride molten salt etching agents, ZnCl_2_ shows the lowest calculated Gibbs free energy, suggesting its superior thermodynamic feasibility for reacting with indium (Table S4, Supporting Information). Furthermore, we hypothesize that only Cl groups are introduced onto the TiB surface during ZnCl_2_ etching, as the reaction occurs in a vacuum environment.^[^ ^21^ ^]^ To mimic this, we use a simplified reaction (Ti_2_InB_2_+ 2.5 Zn Cl_2_ =  2 TiBCl + 2.5 Zn +  InCl_3_) to scrutinize the dynamic microstructure evolution of MS‐TiBT_x_, and Ti_2_InB_2_ phase development is unveiled by heating the mixture of Ti_2_InB_2_ and ZnCl_2_ (1:10 molar ratio) at 600 °C with various durations. As illustrated in **Figure** **2**a,b, the reaction product transitions from Ti_2_InB_2_ to Ti_2_ZnB_2_ and then to TiBCl as the reaction time are confirmed. After 0.5 h, the product of Ti_2_InB_2_ etching is converted to Ti_2_ZnB_2_ with the appearance of metallic In and Zn phases. With further increasing reaction time to 2.0 h, the Ti_2_ZnB_2_ peaks diminish gradually, and the peaks associated with TiBCl emerge. Initially, the presence of metallic Zn and In is noted. However, the prolonged annealing results in the formation of a highly crystallized TiBCl phase, because the InCl_3_, which shows a 583 °C melting point, can react with ZnCl_2_ to form a Zn‐In alloy at 600 °C.^[^ ^22^ ^]^ Further experimental verification shows that the Zn‐In intermediate phase can be etched by ZnCl_2_ (Figure S9, Supporting Information), and the phase evolution is further supported by EDS mapping of the 0.5‐h sample. Moreover, the 0.5 h sample in the transition from Ti_2_ZnB_2_ to TiBCl displays a core‐shell structure: the core region is dense and rich in Zn, while the edge region is delaminated along the in‐plane direction and enriched in Cl (Figure 2c).

To further elucidate the etching mechanism, we calculated the Gibbs free energy changes for the interactions between Al or In atoms from the *ort*‐MAB or *h*‐MAB phases and the Lewis salt ZnCl_2_ at various temperatures. This analysis builds upon previous studies of MoB *ort*‐MBene formation through the deintercalation of Al from MoAlB using a ZnCl_2_ molten salt etching approach.^[^ ^23^ ^]^ We also examined the formation of different MBenes (metal borides) or Cl‐MBenes products from the *ort*‐MAB and *h*‐MAB phases, as detailed in the computational methods section. The Gibbs free energy calculation (Figure 2d) indicates that both *ort*‐MAB and *h*‐MAB phases can be successfully etched into their corresponding MBenes using ZnCl_2_ molten salt; the *h*‐MAB phase is more likely to etch into Cl‐MBenes across the temperature range 0 K‐3000 K, while the *ort*‐MAB phase tends to yield MBenes (metal borides) under the same reaction conditions. This finding explains why Ti_2_InB_2_ can convert to TiBCl, whereas Mo_2_AlB_2_ does not easily form Cl‐terminated MBenes.

The A‐layer removal involves both the chemical reactivity of the A element and its in‐plane migration during dissolution (Figure S10, Supporting Information). Therefore, we calculated the energy barrier for the immigration of In or Al atoms between adjacent A sites in the *h*‐MAB and *ort*‐MAB phases. As illustrated in Figure 2e, migrating Al atoms in *ort*‐MAB phases requires overcoming an energy barrier of 1.49–4.96 eV, whereas In migration in *h*‐MAB involves a significantly lower barrier of 0.73–0.93 eV. These results suggest that In atoms in *h*‐MAB phases can be removed more easily than Al atoms in *ort*‐MAB phases from a dynamic perspective.

In summary, our work shows that In layers in the *h*‐MAB phase are more readily and selectively etched compared to the A layers in *ort*‐MAB phases, both from thermodynamic and dynamic perspectives. The A layers in *ort*‐MAB phases, which are predominantly composed of Al, are less amenable to selective etching. This conclusion is consistent with challenges reported in previous experiments.^[^ ^9^, ^19^ ^]^ Therefore, *h*‐MAB phases may be more promising as MBene precursors compared to *ort*‐MAB phases.

### 2.3 Characterizations of the Delaminated TiBT_x_ *h*‐MBene

With the advancement of ultrathin materials, 2D single‐ and few‐layered MXenes have garnered significant interest due to their beneficial properties, such as low ion diffusion barriers, low open‐circuit voltages, and high specific surface areas.^[^ ^24^ ^]^ To investigate similar properties in *h*‐MBene, we propose exfoliating MS‐TiBT_x_ by intercalating TBAOH, followed by sonication to separate the layers. The addition of TBAOH to the MS‐TiBT_x_ suspension creates a pH gradient between the acidic MS‐TiBT_x_ and the alkaline TBAOH electrolyte. This gradient promotes ion exchange between the bulky tetraalkylammonium ions (TBA^+^) and protons, causing the MBene structure swelling.^[^ ^17^ ^]^ After washing, the TBA^+^‐intercalated MBene materials were sonicated in deionized water to promote exfoliation, followed by centrifugation. The resulting supernatant is a grey‐black dispersion of delaminated MS‐TiBT_x_ *h*‐MBene (denoted as d‐TiBT_x_) in water (Figure S11a, Supporting Information). This dispersion exhibited a distinct Tyndall effect (Figure S11b, Supporting Information), confirming the presence of a colloidal suspension.^[^ ^25^ ^]^ However, unlike delaminated HF‐MXene, the d‐TiBT_x_ *h*‐MBene do not form a cohesive film upon filtration (Figure S12, Supporting Information). This challenge can be attributed to the small initial size of *h*‐MAB phase, resulting in smaller flake sizes of d‐TiBT_x_. This issue underscores the need for further optimization of the exfoliation process and development toward potential applications in flexible energy storage devices.

**Figure 3:**
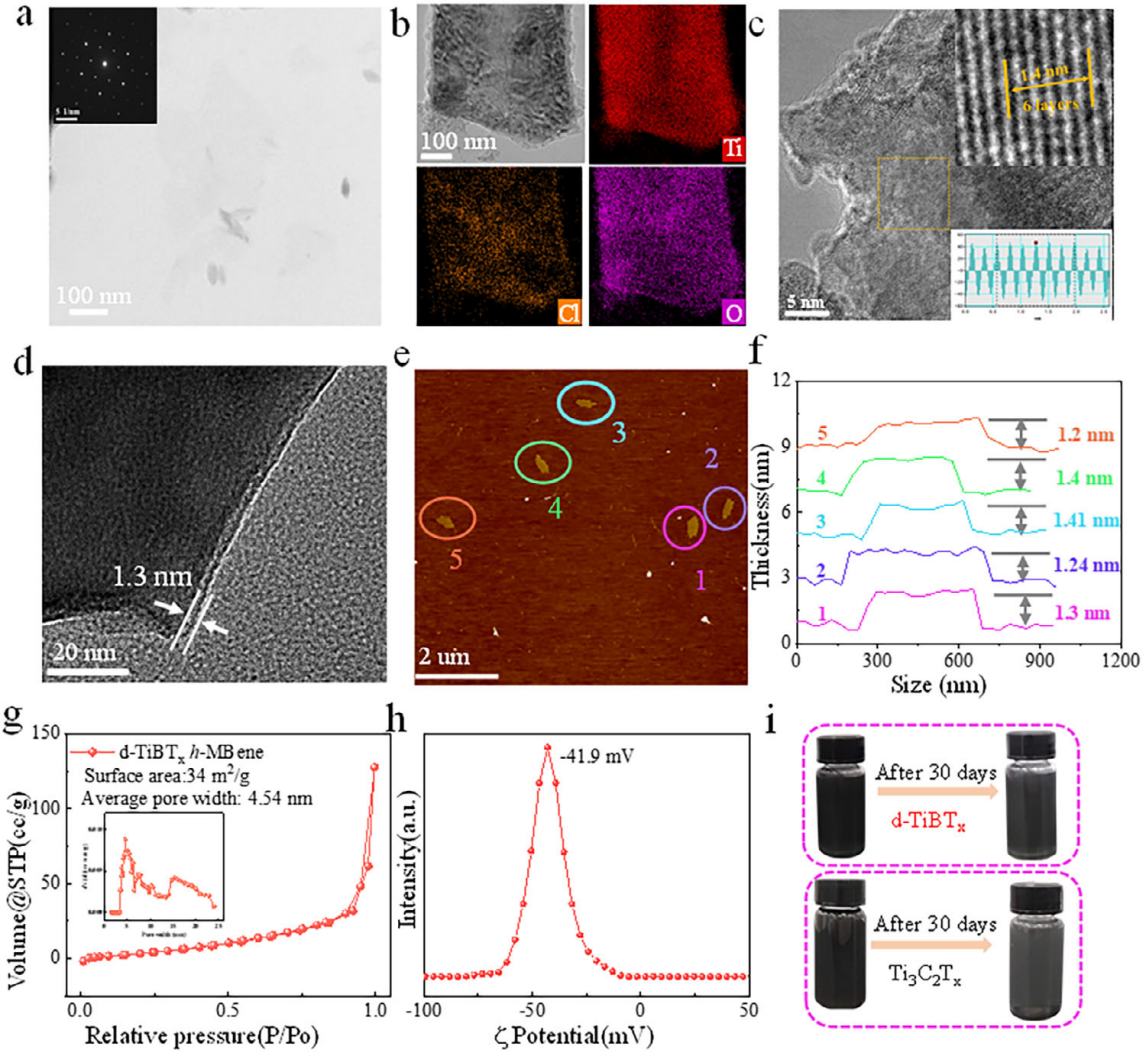
Structural characterization of 2D delaminated TiBT_x_ *h*‐MBene flakes. a) TEM image with inset SAED pattern; b) EDS; c,d) HRTEM; e,f) AFM and its corresponding height profile; g) Nitrogen adsorption‐desorption isotherms and pore size distribution; h) Zeta potential measurement; i) Photographs of d‐TiBT_x_ and Ti_3_C_2_T_x_ dispersions at the initial state and after 30 days at 0 °C.

The d‐TiBT_x_ morphology was characterized via TEM and atomic force microscopy (AFM). TEM image (**Figure** **3**a) reveals that d‐TiBT_x_ are transparent flakes nearly, with a hexagonal crystal system implied by SAED pattern (inset of Figure 3a), confirming that the crystallinity of d‐TiBT_x_ *h*‐MBene nanosheets can be preserved under TBAOH treatment. EDS mapping images (Figure 3b; Figure S13a,b, Supporting Information) identify Ti, B, Cl, and O elements in the d‐TiBT_x_, while In element is almost undetectable, indicating successful etching and the ‐Cl and ‐O terminations presence. HRTEM d‐TiBT_x_ (Figure 3c) exhibits lattice fringes of 0.23 nm, corresponding to the (100) planes of d‐TiBT_x_. The HRTEM result in Figure 3d confirms that the thickness is ≈1.3 nm, equivalent to ≈2 layer thick considering the monolayer thickness of TiBCl (≈0.65 nm). AFM characterization further supports the ultrathin nature of d‐TiBT_x_ flakes (Figure 3e; Figure S14, Supporting Information), showing that d‐TiBT_x_ has a flat surface with 1.3 nm average thickness of line scan (Figure 3f). The average lateral size of d‐TiBT_x_ *h*‐MBene flakes is estimated to be 130–180 nm, smaller than the ≈2 or ≈1 µm flakes produced by HF‐etching or LiF/HCl etching methods,^[^ ^26^, ^27^ ^]^ respectively. However, the Brunauer–Emmett–Teller (BET) surface area increased from 28 to 34 m^2^/g following TBAOH and sonication treatment (Figure 3g). The filtered d‐TiBT_x_ nanosheets exhibit a relatively low conductivity of 196 S m^−1^, which is comparable to the first reported multilayer Ti_3_C_2_ MXenes.^[^ ^20^ ^]^ However, optimized MXenes have currently achieved conductivity close to that of graphene through surface functionalization and interfacial engineering,^[^ ^28^, ^29^ ^]^ which suggests that MBene also has the potential to achieve high conductivity.

The d‐TiBT_x_ surface chemistry was further analyzed by XPS. The high‐resolution of Ti 2p XPS spectra (Figure S15a, Supporting Information) reveals the presence of Ti─O and Ti─Cl bonds, indicative of ‐O and ‐Cl surface terminations^11^, and the peaks at 453.7 and 460.4 eV are attributed to Ti─B bonds, characteristic of metal borides.^[^ ^30^ ^]^ The B 1s spectra show two peaks at 191.7 and 187.4 eV, corresponding to B─O and B─Ti bonds,^[^ ^31^ ^]^ respectively (Figure S15b, Supporting Information). The Cl 2*p* and O 1*s* spectra confirm the presence of Cl‐ and O‐terminated surface functional groups on the d‐TiBT_x_ *h*‐MBene (Figure S15c,d, Supporting Information). The Cl groups are expected from the Lewis salt etching process, while O surface groups likely formed during the oxidation treatment in the TBAOH washing process and subsequent sonication. These processes result in the presence of O‐ and Cl‐terminated functional groups on the 2D d‐TiBT_x_ flakes.

The stability of 2D materials is crucial for various applications, such as printing inks and sprayed coatings.^[^ ^31^ ^]^ Notably, previous studies have indicated that Cl‐terminated MXenes exhibit better stability than F‐terminated MXenes.^[^ ^14^, ^22^ ^]^ Given its lack of fluorine, d‐TiBT_x_ is expected to show good stability. This conjecture can be supported by dispersing d‐TiBT_x_ *h*‐MBene into aqueous solution at 0 °C and 25 °C for 45 and 30 days, respectively. As shown in Figure S16 (Supporting Information), the 25 °C dispersion becomes transparent in the upper layer after 15 days, with sediment formation and near‐total clearing of the supernatant by 30 days, suggesting oxidation due to dissolved O_2_.^[^ ^32^ ^]^ In contrast, the d‐TiBT_x_ with 0 °C suspension exhibits no significant changes after 30 days, with only slight color lightening observed after 45 days. This stability is notably superior to that of HF‐MXenes (Figure 3i), which typically show significant degradation in aqueous solutions at room temperature within ≈15 days.^[^ ^33^ ^]^ Also, the zeta potential (ζ) of d‐TiBT_x_ suspensions in water is surveyed for assessing the surface charge and particle interactions. As seen, the suspension exhibited a zeta potential of ≈–41.9 mV (Figure 3h,), which is higher than that of the reported Ti_3_C_2_T_x_ (−29 mV),^[^ ^34^ ^]^ suggesting that d‐TiBT_x_ has significantly better dispersion stability compared to HF‐Ti_3_C_2_T_x_. Additionally, the particle size of delaminated d‐TiBT_x_ flakes (Figure S17, Supporting Information) focuses on 160 nm in consistent with AFM measurement.

### 2.4 Electrochemical Performance

**Figure 4:**
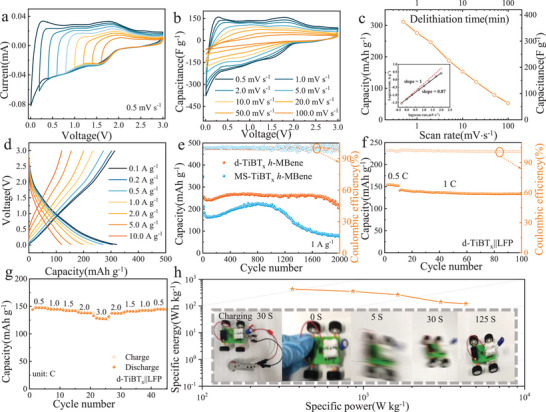
Electrochemical performance of TiBT_x_ *h*‐MBene anodes for LIBs. a) CV profiles of d‐TiBT_x_ *h*‐MBene with different cut‐off potentials; b) CV profiles of d‐TiBT_x_ h‐MBene with different scan rate; c) Variation of capacity and capacitance as function of scan rate in the d‐TiBT_x_ electrode; d) Galvanostatic charge‐discharge (GCD) profiles of d‐TiBT_x_; e) Long‐term cyclability of MS‐TiBT_x_ and d‐TiBT_x_; f) Cycling performance of d‐TiBT_x_||LFP full battery at 1.0 C and g) measured rate capabilities; h) Ragone plot for the d‐TiBT_x_||LFP full‐cell (inset: an electronic toy car powered by a single d‐TiBT_x_||LFP LFP full‐cell operating normally).

Due to the remarkable success of MXenes in energy storage and ion transport, significant efforts have recently focused on the theoretical study of 2D MBenes.^[^ ^35^, ^36^, ^37^ ^]^ These calculations suggest that MBenes could perform exceptionally well as anodes in ion batteries.^[^ ^38^, ^39^ ^]^ To evaluate the practical performance of synthesized TiBT_x_ as battery electrodes, the Li^+^ storage properties of electrodes made from MS‐TiBT_x_ and d‐TiBT_x_ were investigated. Figure S18a (Supporting Information) shows the first three cyclic voltammetry (CV) cycles of d‐TiBT_x_ anode. During the initial cycle, an irreversible capacity loss occurs during the reduction (lithiation) process, attributed to the formation of the solid electrolyte interphase (SEI) layer.^[^ ^40^, ^41^, ^42^ ^]^ In the following cycle, CV measurements exhibit a rectangular‐like shape with no redox peaks during Li intercalation/deintercalation reactions. A similar behavior is observed for MS‐TiBT_x_ *h*‐MBene (Figure S18b, Supporting Information). The absence of redox peaks in the rectangular CV profile indicates a pseudocapacitive energy storage mechanism for TiBT_x_ *h*‐MBenes, which is further supported by the consistency of d‐TiBT_x_ CV profiles recorded with different negative cut‐off potentials at 0.5 mV s^−1^ scan rate (**Figure** **4**a).

To assess charge storage kinetics, the relationship between electrochemical current (*i*) and scan rate (*v*) was analyzed. Theoretically, the current follows the scan rate as *i∼ v^b^*, where a *b*‐value of 1 indicates a fully capacitive process, and a *b*‐value of 0.5 corresponds to battery‐type storage behavior.^[^ ^43^ ^]^ The CV profiles of d‐TiBT_x_ *h*‐MBene recorded at scan rates from 0.5 to 100 mV s^−1^ are presented in Figure 4b. The specific lithiation capacities and capacitances, as a function of charge‐discharge times and scan rates calculated from these CV profiles, are plotted in Figure 4c. The inset of Figure 4c shows a logarithmic plot of *i* versus *v*, revealing a linear relationship with a slope of *b* = 0.87 for scan rates between 0.5 and 100 mV s^−1^. This slope indicates a capacitive‐like charge storage behavior for d‐TiBT_x_ *h*‐MBene. For d‐TiBT_x_ electrode, the discharge capacity increased along with the cycle number, specifically from ≈320 mAh g^−1^ in the first cycle to ≈530 mAh g^−1^ after 500 cycles, then stabilized upon the subsequent cycling process (Figure S19, Supporting Information). This increase is attributed to electrochemical activation, a phenomenon commonly observed in MBene‐based anodes.^[^ ^6^, ^10^ ^]^ Additionally, Figure 4(d) presents the charge/discharge voltage profiles for d‐TiBT_x_ electrode, which achieves a maximum 120 mAh g^−1^ at 10 A g^−1^. This performance exceeds the highest capacity values previously reported for MXenes, highlighting its superior electrochemical capabilities (Table S5, Supporting Information). In contrast, the MS‐TiBT_x_ *h*‐MBene shows a capacity of 270 mAh g^−1^ at 0.1 A g^−1^ and 60 mAh g^−1^ at 10 A g^−1^ (Figure S20, Supporting Information). The d‐TiBT_x_ *h*‐MBene exhibits a capacity retention of 37.5% when transitioning from 0.1 to 10 A g^−1^, highlighting its high‐power capability. In comparison, the MS‐TiBT_x_ *h*‐MBene retains only 22.2% of its capacity over the same current density range.

Furthermore, the d‐TiBT_x_ *h*‐MBene electrode maintains a stable capacity of 202 mAh g^−1^ over 2000 cycles at 1 A g^−1^, with a high‐capacity retention of 77.69% relative to the peak value of 260 mAh g^−1^ observed at the 300th discharge cycle (Figure 4e). On the other hand, the MS‐TiBT_x_ *h*‐MBene electrode shows a gradual increase in capacity, reaching a maximum of 223 mAh g^−1^ by the 600th cycle and stabilizing at a reversible specific capacity of ≈210 mAh g^−1^ after 1000 cycles. These electrochemical results highlight the exceptional performance of the synthesized d‐TiBT_x_ *h*‐MBene electrode. In addition, SEM images (Figure S21, Supporting Information) of d‐TiBT_x_ *h*‐MBene electrode after 1000 cycles at 1 A g^−1^ in LIBs still retain nanosheet‐like morphology, indicating the superior stability of d‐TiBT_x_ electrode material over extensive cycling. To investigate the in‐cell dynamics during the discharge‐charge cycle, operando EIS studies were performed on the d‐TiBT_x_ electrode in LIBs at various specific voltages.^[^ ^44^ ^]^ The EIS plots recorded at different bias potentials (Figure S22a,b, Supporting Information) show a charge‐transfer resistance of ≈50 Ω cm^2^, accompanied by restricted diffusion behavior, as evidenced by a sharp increase in the imaginary part of the impedance at low frequencies.^[^ ^45^ ^]^

Taking for the excellent electrochemical properties of d‐TiBTx, its practical applications are explored by assembling Li‐ion full‐cells with d‐TiBT_x_ anode and commercial LiFePO_4_ (LFP) cathode. On the charge/discharge capacities of d‐TiBT_x_ and LFP at a constant current of 0.1 A g^−1^ (Figure S23a, Supporting Information), the optimized mass loading ratio of the anode to cathode was determined to be 1:2 for the d‐TiBT_x_||LFP full‐cell. The cell operated within a voltage window of 2.0–4.0 V versus Li/Li^+^. Long‐term stability tests conducted at 1.0 C demonstrated excellent cycling performance, with a capacity retention of 94.3% after 100 cycles and ≈100% coulombic efficiency (Figure 4f). As seen, the rate capability was outstanding with the full‐cell delivering a high specific capacity of 147 mAh g^−1^ at 0.5 C and 127 mAh g^−1^ at 3.0 C (Figure 4g; Figure S23b, Supporting Information). Remarkably, when the current density was reverted to 0.5 C after 40 cycles, the capacity recovered to 145 mAh g^−1^. Also, the d‐TiBT_x_||LFP full‐cell exhibited impressive energy and power densities, achieving a maximum energy density of 425 Wh kg^−1^ at 363 W kg^−1^ and a high‐power density of 4321 W kg^−1^ at 121 Wh kg^−1^. This energy output was sufficient to power a toy car (Figure 4h), demonstrating its practical feasibility. These results highlight the potential of d‐TiBT_x_||LFP full‐cells for real‐world applications, confirming their high capacity, excellent rate performance, and remarkable stability in lithium‐ion batteries.

The enhanced power capability of the d‐TiBT_x_ can be attributed to its increased electrochemically accessible specific surface area (Figure 3g) and faster ion transport. EDS and XPS analysis of the d‐TiBT_x_ reveals that the primary effect of the TBAOH treatment is an increase in the –O content (Tables S6 and S7, Supporting Information), ascribable to the dissolved‐oxygen exposure during the sonication process. Therefore, further studies are needed to understand how the increased –O content affects ion transport and to confirm the storage mechanism of Li⁺ in the d‐TiBT_x_ *h*‐MBene.

### 2.5 Storage Mechanism of Li^+^ in TiBT_x_ *h*‐MBene

To gain deeper insights into the Li⁺ storage mechanism in TiBT_x_ *h*‐MBene, the DFT calculations with experimental studies were performed to reveal the adsorption and diffusion behavior of Li atoms on 2D TiBT_x_ surfaces, focusing on the evolution of surface functional groups. Figure S24 (Supporting Information) shows models of TiB surfaces functionalized with varying amounts of ‐Cl and ‐O groups. Previous reports have demonstrated that metal vacancies are likely to form in MX/Benes,^[^ ^46^, ^47^ ^]^ but the evaluated vacancy formation energies of four types with varying coverage indicate that defects are not easily formed form on TiBT_x_ MBene (Figure S25, Supporting Information). Therefore, the impact of vacancies is not considered in the subsequent study.

**Figure 5:**
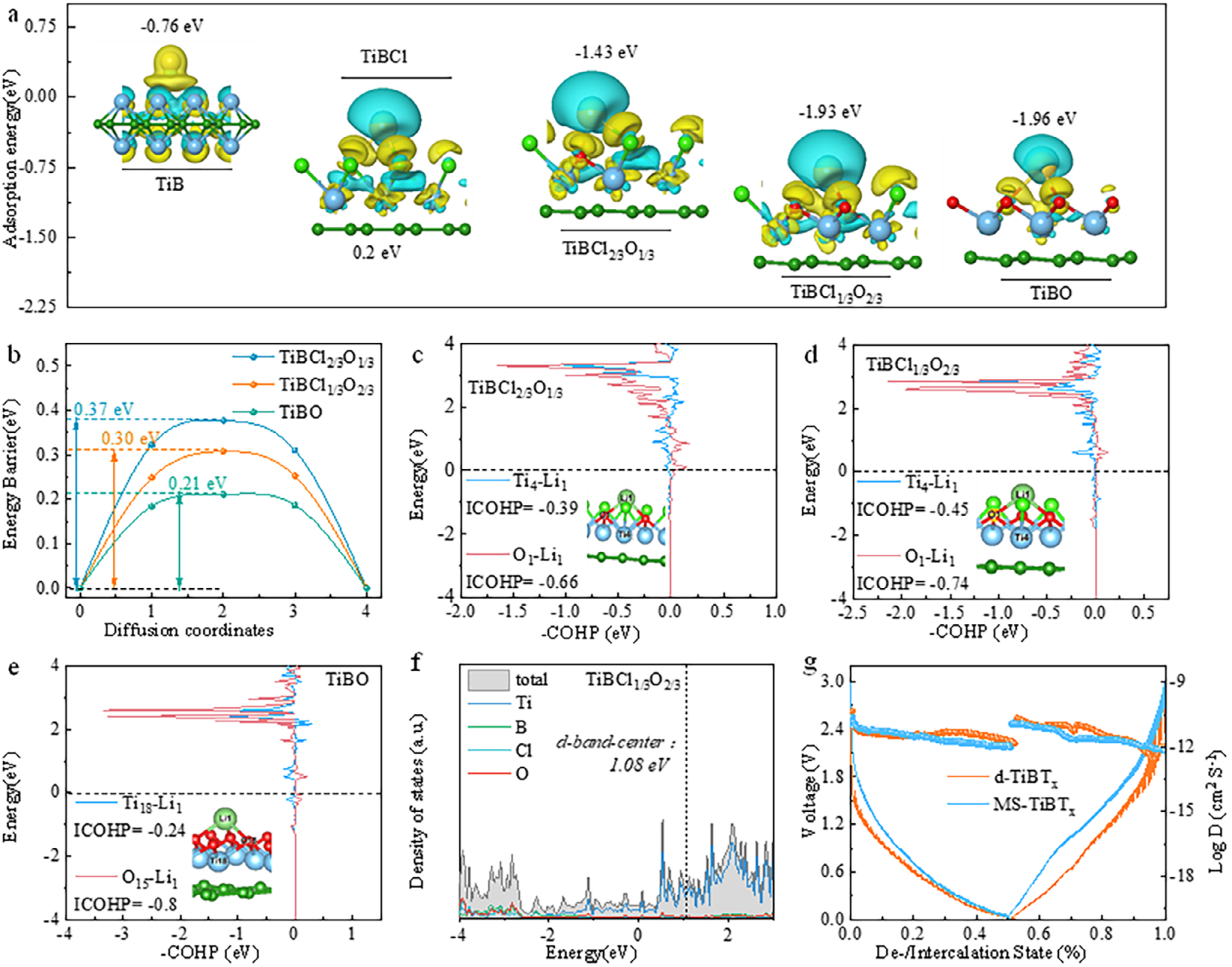
Adsorption and diffusion behavior of Li on 2D TiBT_x_. a) Li^+^ adsorption energy and charge density difference; b) calculated energy barriers for Li migration; c) COHP between Ti/O and Li atoms of Li after adsorption on TiBCl_2/3_O_1/3_, d) TiBCl_1/3_O_2/3_and e) TiBO; f) total DOS of TiBCl_1/3_O_2/3_; the g) Li‐ion diffusion coefficient during charge/discharge.

DFT calculations reveal interesting trends in adsorption energies and diffusion energy barrier of Li atom on these functionalized TiB surfaces (**Figure** **5**a,b; Figure S26, Supporting Information). As the concentration of O‐functional groups increases, the adsorption energies of Li become more negative. Li atoms exhibit negative adsorption energies of −0.76 eV on *h*‐TiB, while *h*‐TiBCl shows positive adsorption energies (0.2 eV) toward of Li atoms, indicating that the chlorinated surface is inferior to Li adsorption. As the TiBCl surface undergoes slight oxidation to form TiBCl_2/3_O_1/3_, the Li adsorption energy becomes significantly more negative, reaching –1.43 eV. Further oxidation to TiBCl_1/3_O_2/3_ results in more negative adsorption energies (−1.93 eV), closely matching those on fully oxidized *h*‐TiBO (−1.96 eV). Furthermore, the simulation results of Ab Initio Molecular Dynamics (AIMD) indicated that Li‐adsorbed configuration in the TiBCl_1/3_O_2/3_ structure is thermally stable (Figure S27, Supporting Information). The diffusion energy barrier for Li atom between neighboring low‐energy adsorption sites are further depicted in Figure S28 (Supporting Information) and Figure 5b. As shown, TiBO exhibits the lowest energy barriers (0.21 eV) than that of TiBCl_1/3_O_2/3_ (0.30 eV) and TiBCl_2/3_O_1/3_ (0.37 eV), suggesting that more oxidized TiB *h*‐MBene could achieve high charge‐discharge rates.

To analyze the low diffusion barrier, the integrated crystal orbital Hamilton population (ICOHP) and d‐band center are obtained.^[^ ^48^, ^49^ ^]^ Figure 5c–e and Figure S29 (Supporting Information) show that the ICOHP values for Li─O and Li─Ti bonds is variable on different functionalized *h*‐MBene surfaces, comparable to those observed for metallic bonding.^[^ ^50^ ^]^ The moderate –ICOHP values suggest that the *h*‐MBene surface can stably absorb Li ions while maintaining a bond strength that is not too high. This balance prevents difficulties with Li ion desorption. Specifically, the calculated –ICOHP values for Li─O and Li─Ti bonds on TiBCl_1/3_O_2/3_ are 0.74 and 0.45 eV, respectively, which are higher than those on TiBCl (0.27 and 0.35 eV) and TiBCl_2/3_O_1/3_ (0.66 and 0.39 eV). This indicates that the bonding strength of Li─O and Li─Ti bonds increases with a higher concentration of O‐functional groups on the *h*‐MBene surface. The total density of the states (TDOS) was analyzed to elucidate the electronic structure of TiBT_x_. As shown in Figure 5f and Figure S30 (Supporting Information), all models show metallic features with the TDOS crossing over the Fermi level, which facilitates charge transport during electrode reactions. Notably, the d‐band center values were calculated to be 0.532 eV for TiB, 0.85 eV for TiBCl, 0.96 eV for TiBCl_2/3_O_1/3_, 1.08 eV for TiBCl_1/3_O_2/3,_ and 1.03 eV for TiBO. According to the d‐band theory proposed by Nørskov and co‐workers,^[^ ^51^ ^]^ the shift in the d‐band center indicates that the introduction of O and/or Cl terminations into TiBT_x_, particularly in TiBCl_1/3_O_2/3_ and TiBO, enhances its Li adsorption capability compared to pristine TiB^[^ ^52^ ^]^ This further supports the substantial impact of oxidation on improving the electrochemical performance.

To investigate the influence of the oxygen‐rich TiB surface on the composition of the SEI layers, XPS spectra of MS‐TiBT_x_ and d‐TiBT_x_ electrodes were collected after the first and 50th cycles (Figure S31, Supporting Information). A Li_2_O‐rich SEI layer, formed on the d‐TiBT_x_ electrode with fast ion diffusion kinetics, significantly enhances lithium‐ion storage performance.^[^ ^53^, ^54^ ^]^ Diffusion coefficients of Li ions (*D_Li⁺_*) on d‐TiBT_x_ were measured using galvanostatic intermittent titration (GITT). The d‐TiBT_x_ electrode demonstrated faster ion diffusion, with *D*_Li⁺_ ranging of 10^−10.37^‐10^−9.91^ cm^2^ s^−1^, compared to significantly lower values in the MS‐TiBT_x_ electrode (10^−12.06^‐10^−10.62^ cm^2^ s^−1^) (Figure 5g). Additionally, the d‐TiBT_x_ electrode exhibits a markedly smaller high‐frequency semicircle compared to the pristine MS‐TiBT_x_ electrode, reflecting a lower charge‐transfer resistance (*R_ct_*). Furthermore, with increasing cycle number, the semicircle radius of both electrodes gradually decreases (Figure S32, Supporting Information). The d‐TiBT_x_ electrode shows a more pronounced reduction in impedance, confirming the enhanced transport of both ions and electrons within the electrode.

The above results uphold that TiBCl_1/3_O_2/3_ monolayer is promising for Li energy storage. Strikingly, the theoretical specific capacity (Figure S33a–d, Supporting Information) of TiBCl_1/3_O_2/3_ for Li ions was calculated to be 1153 mAh g^−1^ (Ti_18_B_18_Cl_6_O_12_Li_108_), and the calculated open‐circuit voltage (OCV) for intercalation reactions involving Li^+^ ions on the TiBCl_1/3_O_2/3_ surface was estimated to be as low as 0.38 V (Figure S33e, Supporting Information), which are comparable to the graphite (≈0.2 V for Li^+^).^[^ ^55^ ^]^ It should be noted that because of the idealized monolayer assumptions in theoretical models, these calculated values of Li storage of TiBCl_1/3_O_2/3_ are higher than experimental results (530 mAh g^−1^), even though the experimentally prepared d‐TiBT_x_ composition (Ti_1.22_B_1.08_Cl_0.27_O_1.31_, Table S4, Supporting Information) closely resembles the calculated TiBCl_1/3_O_2/3_ model.

## 3 Conclusion

To summarize, we represent the first synthesis of ultrathin TiBT_x_ *h*‐MBene materials using earth‐abundant titanium. With O‐ and Cl‐terminations, the delaminated structure significantly increases the surface area and enhances oxidation, thereby improving the accessibility of active sites and facilitating Li ion transport. These features make that d‐TiBT_x_ as in Li‐ion battery anodes, show impressive electrochemical performance, with specific capacities of 530 mAh g^−1^ at 0.1 A g^−1^ and excellent rate capabilities of 120 mAh g^−1^ at 10 A g^−1^. Moreover, the assembled d‐TiBT_x_||LFP full‐cell exhibits a maximum energy density of 425 Wh kg^−1^ at 363 W kg^−1^ and capacity retention of 94.3% after 100 cycles, which can make an electronic toy car run normally. Our results highlight that the ultrathin *h*‐MBenes are available for a wide range of energy storage devices, including non‐aqueous batteries and capacitors. Furthermore, this study provides new theoretical and experimental insights into the dynamic reaction mechanisms of molten salt etching in MAX/MAB phases, contributing to the development of more effective etching strategies. By using abundant and less harmful elements and enhancing the performance of energy storage devices, our findings contribute meaningfully to the sustainability of future electrochemical systems, offering a pathway toward greener and more efficient energy storage solutions 4.

## 4 Experimental Section

### Preparation of MS‐TiBT_x_ *h*‐MBene

Ti_2_InB_2_ MAB phase was synthesized as previously reported,^[^ ^11^ ^]^ and Zinc chloride (anhydrous, ZnCl_2_, > 98 wt.% purity) was purchased. For the synthesis of MS‐TiBT_x_ *h*‐MBene, Ti_2_InB_2_ and ZnCl_2_ were mixed uniformly in a mortar under an argon atmosphere inside a glovebox and subsequently cold‐pressed into a 15 mm diameter disk. The disk was sealed in a vacuum quartz tube, followed by a reaction at 600 °C for 8 h. After the reaction, residual ZnCl_2_ was removed by washing the product with deionized water, and the resulting material was dried overnight in a vacuum oven at 50 °C.

### Exfoliation and Delamination of MS‐TiBT_x_ *h*‐MBene

A total of 0.2 g of MS‐TiBT_x_ *h*‐MBene was introduced into 10 ml of a 40 wt.% aqueous TBAOH solution under an Ar atmosphere and stirred at room temperature for 24 h. Afterward, the mixture was centrifuged and washed with deionized water. The resulting sediment was dispersed in 40 ml of water and sonicated under Ar bubbling for ≈6 h at low temperatures (using an ice bath). Subsequently, the mixture was centrifuged at 3500 rpm for 20 min to remove the sediment. The supernatant containing delaminated TiBT_x_ *h*‐MBene (d‐TiBT_x_ *h*‐MBene) (T_x_ denotes ‐Cl and ‐O terminations) was further centrifuged at 10 000 rpm for 60 min, and the final product was obtained through freeze‐drying.

### Physical Characterization

X‐ray diffraction (XRD) analysis was conducted using a D4 Endeavor diffractometer (Bruker, Germany) with Cu Kα radiation (λ = 0.154 nm). The material's morphology was examined using a JSM 7100F scanning electron microscope (SEM) (JEOL, Japan). Transmission electron microscopy (TEM) and high‐resolution transmission electron microscopy (HRTEM) images were acquired with a JEM‐2100F microscope operated at 200 kV. Elemental mapping was performed using energy‐dispersive X‐ray spectroscopy (EDS), though it should be noted that boron (B) is too light to be detected by EDS.^[^ ^56^ ^]^ X‐ray photoelectron spectroscopy (XPS) was conducted using an ESCALAB system with an Mg‐Kα light source. Scanning transmission electron microscopy (STEM) and electron energy loss spectroscopy (EELS) analyses were performed on an FEI Themis Z microscope operating at 80 kV. Atomic force microscopy (AFM) measurements were carried out with a Bruker Multimode 8 in ScanAsyst mode. Zeta potential was determined using a Zetasizer Nano ZS90 (Malvern Instruments Ltd., UK). Inductively coupled plasma (ICP) analysis was performed using an Agilent ICP‐OES 730 system. The specific surface area of each sample was determined using the multi‐point Brunauer‒Emmett‒Teller (BET) method.

### Electrochemical Measurements

Electrochemical tests were conducted using CR2032 coin‐type cells, assembled in an Ar‐filled glovebox with H_2_O and O_2_ levels maintained below 1 ppm. Anodes were prepared by mixing active material (70 wt.%), carbon black (20 wt.%), and polyvinylidene fluoride (10 wt.%) in 1‐methyl‐2‐pyrrolidone (NMP) to form a homogeneous slurry. The slurry was coated onto copper foil and dried in a vacuum at 60 °C overnight. The active material mass loading was ≈1.0–1.2 mg cm^−2^. For LIBs, lithium foil, 1 M LiPF_6_ in a 1:1 (v/v) mixture of ethylene carbonate (EC) and diethyl carbonate (DEC), and Celgard 2400 were used as the counter electrode, electrolyte, and separator, respectively. Full cells were assembled using a prelithiated d‐TiBT_x_ anode and a LiFePO_4_ (LFP) cathode. Prelithiation of the d‐TiBT_x_ anode was achieved after five cycles at 0.1 A g^−1^ in a half‐cell configuration. The N/P ratio between d‐TiBT_x_ and LFP was set to ≈1.2:1. The performance of the full cells was evaluated within a voltage range of 2.0–4.0 V (based on LFP and d‐TiBT_x_). Additionally, all electrochemical measurements were conducted at 25 °C. The energy density value was calculated from the full cell based on the weight of cathode and anode electrode materials without considering the weight of the electrolyte, current collector, separator, and case in the cell's configuration. The energy density is calculated based on the following equations:

(1) $$E_{g}=\frac{U\times C}{\sum m_{i}}$$

where *E_g_* Is the gravimetric energy density, *U* is the average voltage of the cell, *C* is the areal capacity of the cell, ∑*m_i_* Is the weight of the cathode and anode material.

Cyclic voltammetry and electrochemical impedance spectroscopy were performed using a CHI660E electrochemical workstation. Galvanostatic charge/discharge (GCD) tests were conducted with a NEWARE CT‐4008Tn battery test system (Shenzhen, China). Both cyclic voltammetry and galvanostatic cycling were carried out within a potential window of 0.01 to 3 V. Electrochemical impedance spectroscopy measurements were taken at open circuit potential with a 10 mV amplitude, covering a frequency range from 0.01 Hz to 100 kHz.

### Computational Methods

First‐principles calculations were carried out using density functional theory (DFT) within the projector augmented wave (PAW) method^[^ ^57^ ^]^ implemented in the Vienna Ab initio Simulation Package (VASP).^[^ ^58^ ^]^ The Perdew–Burke–Ernzerhof (PBE) version of the generalized gradient approximation (GGA)^[^ ^59^ ^]^ was used to address the exchange and correlation energies. A plane‐wave cutoff energy of 520 eV was employed to ensure the convergence of the total energy. To avoid interactions between two neighboring layers, a large vacuum space of 20 Å was set along the c‐axis for all of the *h*‐MBene calculations. The A elements' in‐plane migration barriers were explored using the climbing‐image nudged elastic band (CI‐NEB) method.^[^ ^60^ ^]^ The adsorption energies (*E*_b_) of Li atoms on various surfaces were determined using the following equation:

(2) $$E_{b}=E_{\mathrm{Li}@\mathrm{surf}}-E_{\mathrm{surf}}-E_{\mathrm{Li}}$$

where *E*_Li@surf_, *E*_surf_ and *E*_Li_ are the calculated energies of Li@surface, bare surface, and a Li atom in the most stable Li metal phase, respectively.

The detailed calculation method of the theoretical capacity. A 3 × 3 supercell of TiBCl_1/3_O_2/3_ monolayer was constructed. These atomic layers were separated from adjacent layers by a vacuum thickness of 50 Å along the Z direction. The detailed calculation method is: (1) The average adsorption energy, *E_ave_*, is defined as

(3) $$E_{ave}=\frac{E_{{A_{x}}_{@surface}}-E_{surface}-\left(x\right)E_{A}}{x}\;$$

where *E_surface_* represents the energy of TiBCl_1/3_O_2/3_ monolayer, $E_{{A_{x}}_{@surface}}$ denotes the energy of *x* A Li atoms adsorbed on the TiBCl_1/3_O_2/3_ monolayer, *E_A_* is the energy of each Li atom in bulk, and *x* is the number of A atoms adsorbed. When *E_ave_* Shifts from negative to positive, the adsorbed Li atoms aggregate into clusters due to cohesive energy, and further adsorption ceases. The theoretical capacity, C, is defined as

(4) $$C=\frac{xnF}{M_{surface}+xM_{A}}\;$$

where *x* represents the maximum number of atoms adsorbed, *n* represents the valence of the A species, *F* represents the Faraday constant (26801 mAh g^−1^), and A atoms to the *M_surface_* and *M_A_* represent respective molar weights of TiBCl_1/3_O_2/3_ monolayer and A.

The Open circuit voltage (OCV) is defined as the voltage required to stop the current in a battery. It is equivalent to the adsorption energy of an alkali‐metal ion, divided by the electron charge, and with the opposite sign.

(5) $$OCV=\frac{E_{surface}+\left(x\right)E_{A}-E_{{A_{x}}_{@surface}}}{xe}\;$$

where *E_surface_*, $E_{{A_{x}}_{@surface}}$, *E_A_* And *x* are defined as in Equation (3), and *e* is the electron charge.

The Gibbs free energy was calculated to further confirm the MS‐TiBT_x_ *h*‐MBene material prepared by Lewis salt ZnCl_2_ etching of Ti_2_InB_2_ *h*‐MAB phase. Considering the strong covalent M─B bonding and the weak A layer atoms, as well as the weak interaction of the M‐In and M‐Al in *h*‐MAB/*ort*‐MAB phase, we assume that the M─B bonding remains unchanged, and the Al element and In element can be removed during the etching reactions. The etching reactions can be expressed as:

(6) $$2M_{2}AB_{2}+3\;\mathrm{Zn}\;Cl_{2}=4\;\mathrm{MB}\;+3\;\mathrm{Zn}\;+2\;\mathrm{AC}l_{3}$$

(7) $$M_{2}AB_{2}+2.5\;\mathrm{Zn}\;Cl_{2}=2\;\mathrm{MBCl}+2.5\;\mathrm{Zn}+\mathrm{AC}l_{3}$$

The Gibbs free energies (Δ*G*, eV = KJ mol^−1^/96.4916) between Al or In elements from the *ort*‐MAB or *h*‐MAB phases and Lewis salts ZnCl_2_ (reactions (5) and (6)) were calculated at different temperatures. Note that the negative value of the calculated ΔG indicates the corresponding reaction is thermodynamically spontaneous.

In the ab initio molecular dynamics calculations (AIMD) involving the adsorption of a single Li atom, we used a 3 × 6 supercell. At the same time, in order to avoid periodic interactions, the vacuum layer was set not lower than 20 Å. For geometry optimization, the energy convergence criterion was set to 10–5 eV, and the conjugate gradient method was used to allow complete relaxation of the positions of all atoms until the maximum force experienced by any atom was less than 0.02 eV Å^−1^. The cutoff energy was all set to 500 eV.

## Conflict of Interest

The authors declare no conflict of interest.

## Supporting information



Supporting Information

## Data Availability

The data that support the findings of this study are available from the corresponding author upon reasonable request.
