# Supplementary material for: Engineering Oxygen‐Terminated Hexagonal MBene for Accelerated Lithium Migration and Exceptional Lithium‐Ion Battery Performance
Source: Adv Sci (Weinh). 2025 Sep 26;12(47):e13674. doi: 10.1002/advs.202513674 (PMC12713009; doi:10.1002/advs.202513674)
Supplement: Supplementary file 1 — Supporting Information [file ADVS-12-e13674-s001.docx]

**Supporting Information**

**Engineering Oxygen-Terminated Hexagonal MBene for Accelerated Lithium migration and Exceptional** **Lithium-Ion Battery Performance**

*Qing Shen, LeYang Wang, Pengjie Jiang, Jinming Wang, Yaxin Di, Hui Mei, Junjie Wang*and Laifei Cheng*

* Corresponding author.

E-mail address: wang.junjie0810@gmail.com.

**Supplementary Figures**

**
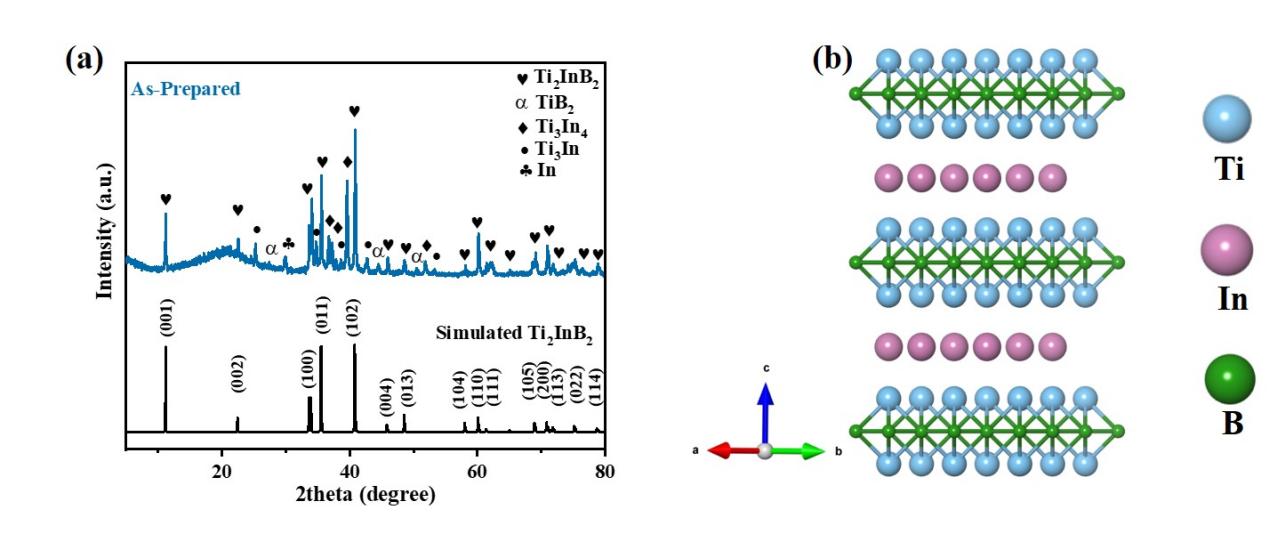
**

**Figure S1:** (a) XRD pattern of the as-prepared Ti_2_InB_2_ *h*-MAB and (b) the corresponding structural diagram.

**
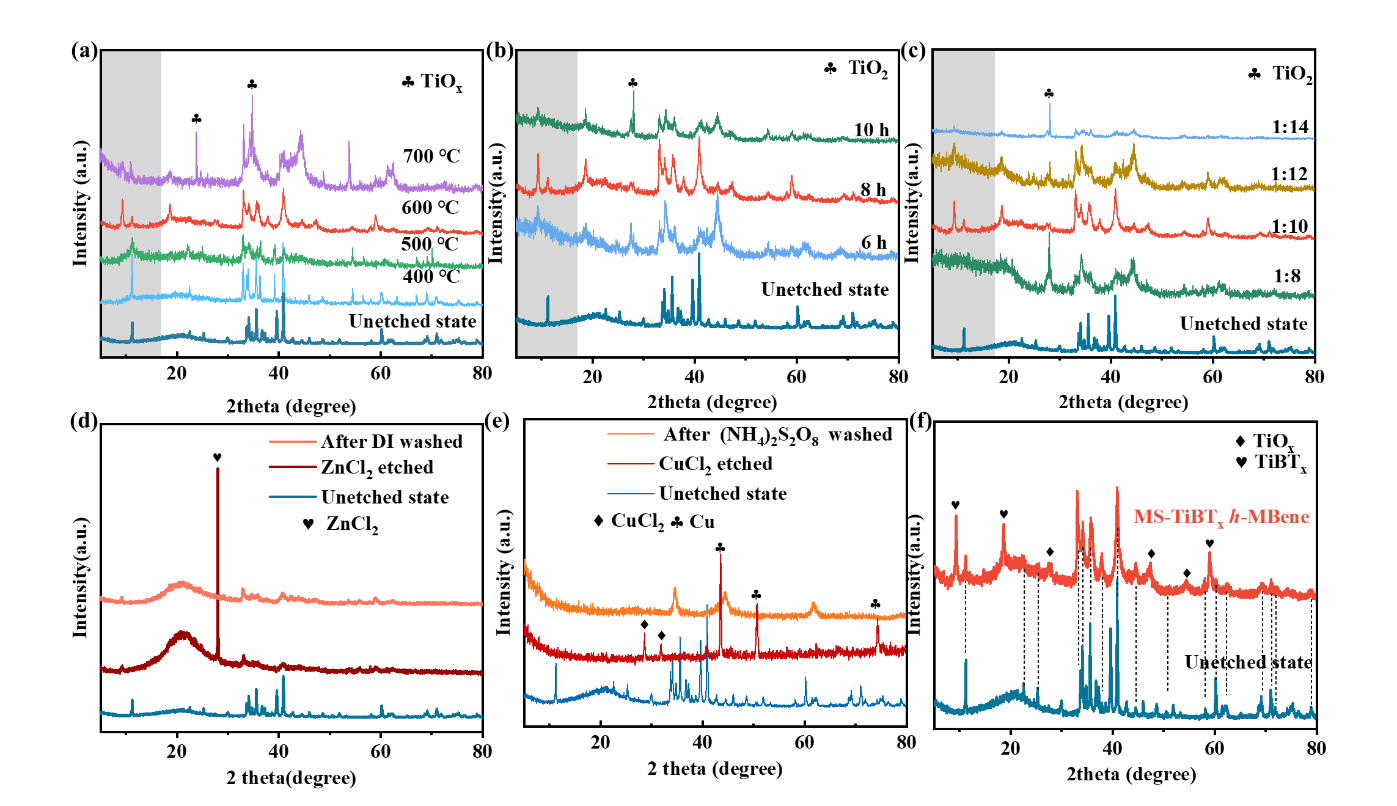
**

**Figure S2:** (a-c) XRD patterns of the etched product using ZnCl_2_ etchant at different temperature, molar ratio and etching time; (d-e) Comparison of the XRD patterns between the raw materials, the initially CuCl_2_/ZnCl_2_ etched products and the product was washed with deionized water; (f) XRD patterns of the raw materials and the MS-TiBT_x_ *h*-MBene.

Compared with the raw (unetched) state, the characteristic peak of (001) shift from 11.19° to 9.36° with different relative intensity at different molar ratio and etching time, corresponding to the different etching degrees. The highest relative intensity of the (00l) peak at 9.36° versus that at 11.19° indicates that etching for 8 h with molar ratio 1:10 is the most suitable condition (Fig S2(a-c)). Besides, the disappeared ZnCl_2_ peak in the XRD pattern after DI washed evidences the successful removal of unreacted ZnCl_2_ salts (Fig S2(d)). compared to CuCl_2_ etching (Fig S2(e)), providing a more efficient etching process.

As presented in Figure S2(f), in the XRD pattern of the MS- TiBT_x_ *h*-MBene, the two strong characteristic peaks corresponding to the raw state are well maintained, demonstrating the good inheritance of structural features from the raw materials^1^. Most of the tiny peaks of byproducts in the etched product from the raw materials, and some minor peaks of byproducts that are present in the raw state are almost invisible in the etched product, indicating that the ZnCl_2_ molten salt etchant can reduce the byproducts derived from the precursor and hence promote the purity of the etched product. Besides, tiny peaks corresponding to TiO_x_ are detected in the etched product, which are mainly attributed to the slight oxidation reaction between the *h*-MAB materials and the residual O_2_ in the pipe furnace during the etching process. It is hard to completely avoid the slight oxidation reaction when using the pipe furnace at a high temperature^2^.


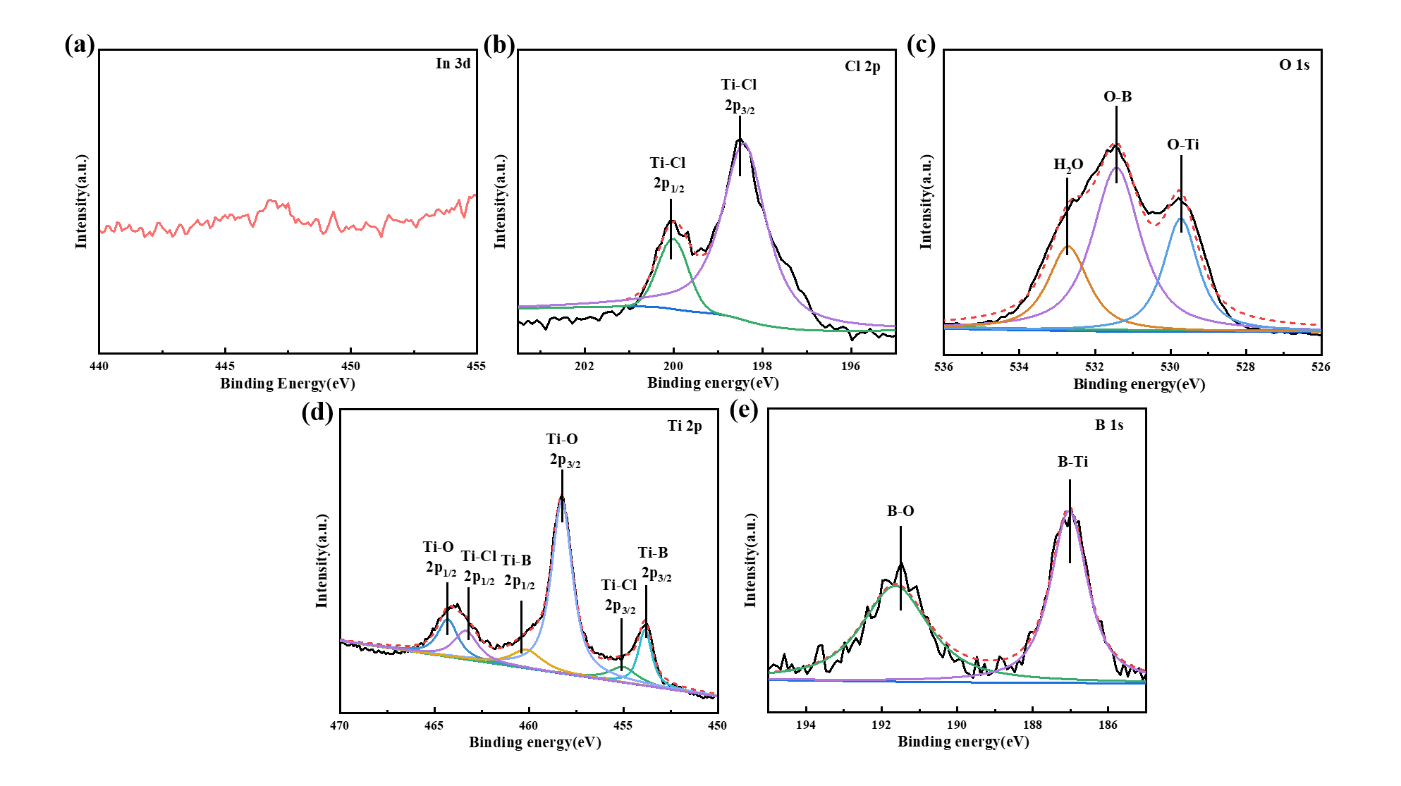


**Figure S3:** High-resolution XPS spectra of MS-TiBT_x_ *h*-MBene (a) In 3d; (b) Cl 2p; (c) O 1s; (d) Ti 2p and (e) B 1s.


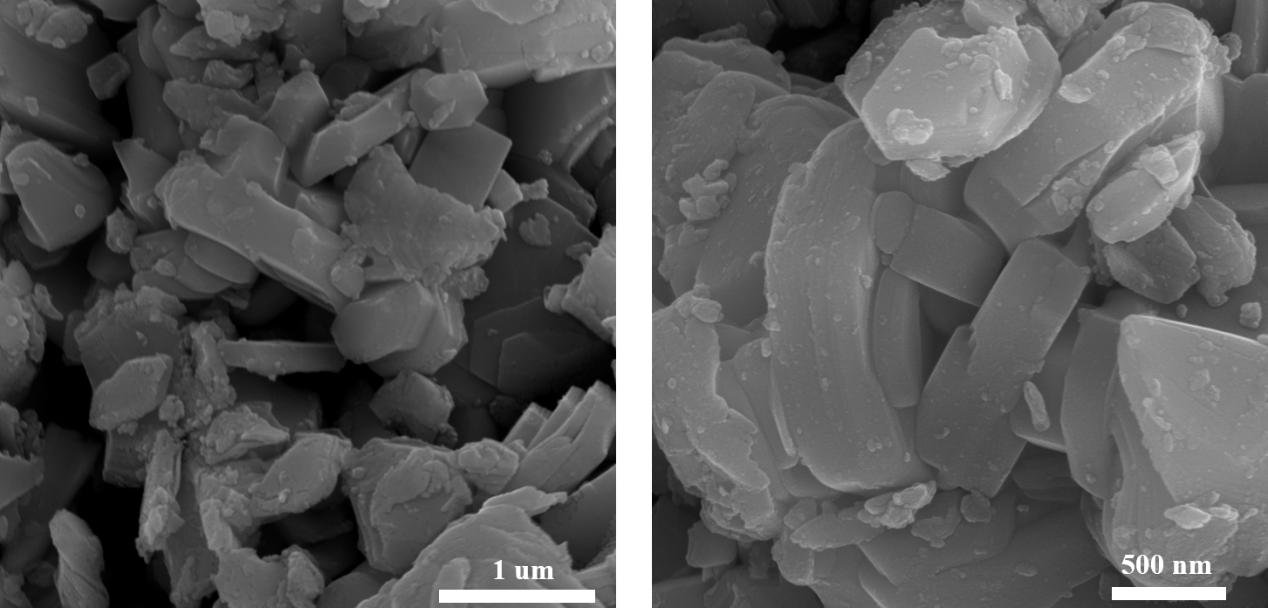


**Figure S4:** SEM images of pristine Ti_2_InB_2_.


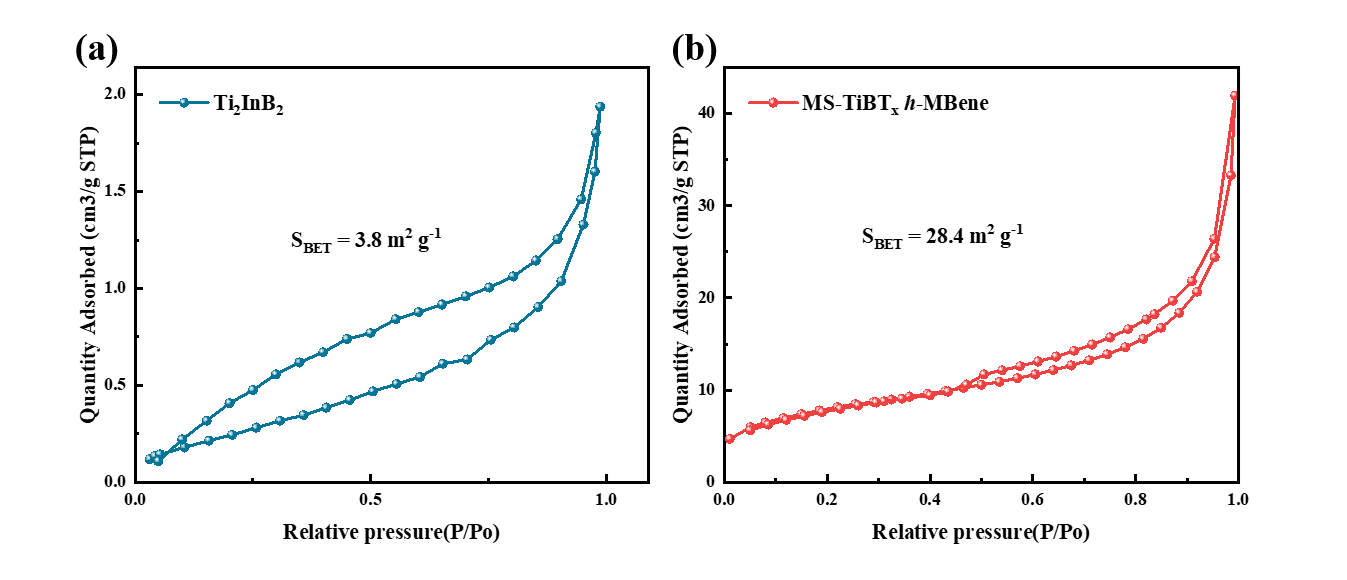


**Figure S5:** N_2_ sorption isotherms of (a) Ti_2_InB_2_ and (b) MS-TiBT_x_ *h*-MBene.

**Figure S6:** (a) Element mapping images of Ti_2_InB_2_ and (b) MS- TiBT_x_ *h*-MBene.

The Element mapping analysis of single platelet clearly reveals the presence of Ti, In, and B components which homogeneously distribute in Ti_2_InB_2_. After ZnCl_2_ treated, In atoms is greatly decreased and O, Cl atoms are dispersed uniformly in MS-TiBT_x_ *h*-MBene, indicating the successful etching.


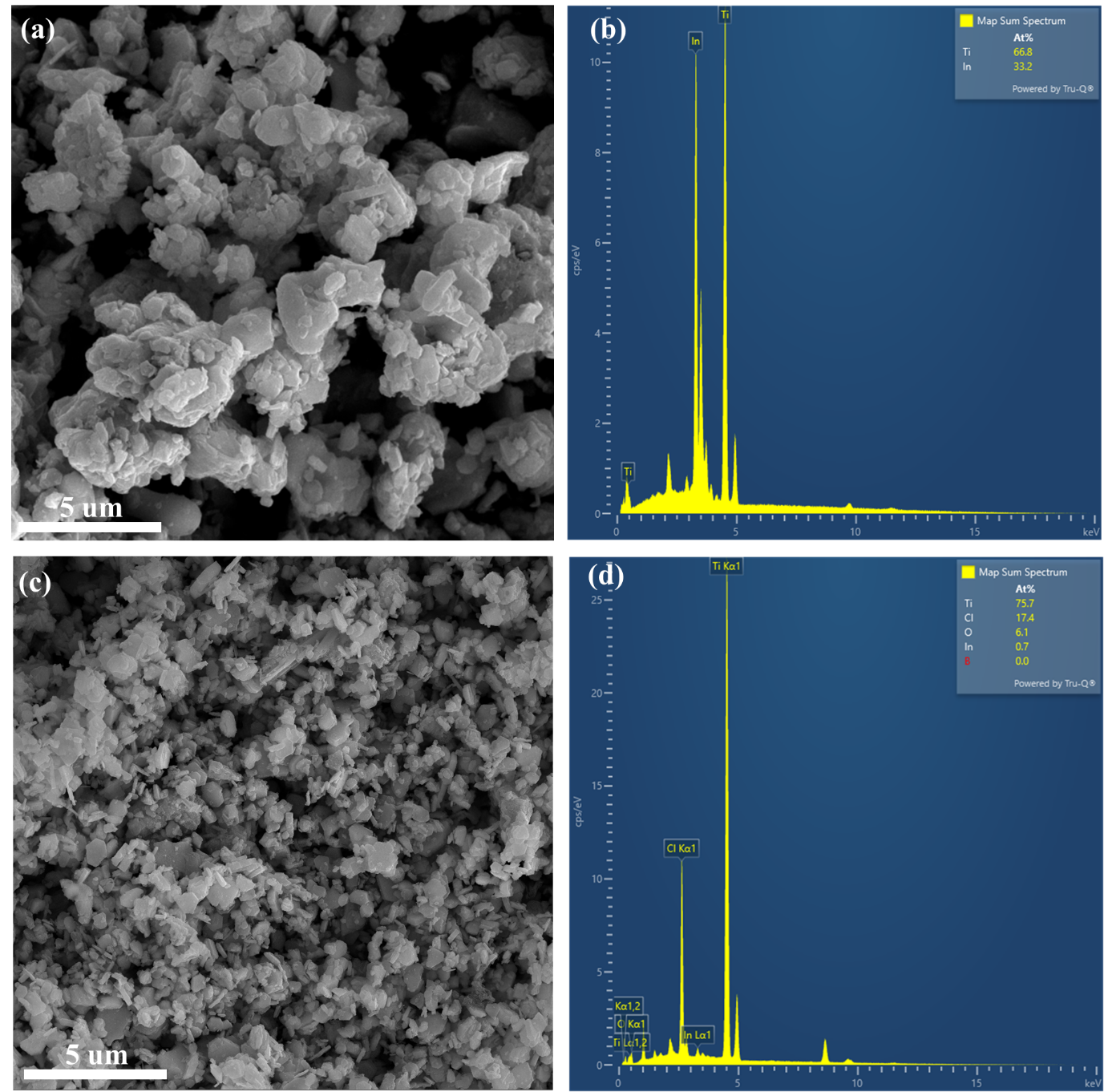


**Figure S7:** EDS results of Ti_2_InB_2_ (a-b) and (c-d) MS-TiBT_x_ *h*-MBene.

The single of In after etching was significantly decreased.


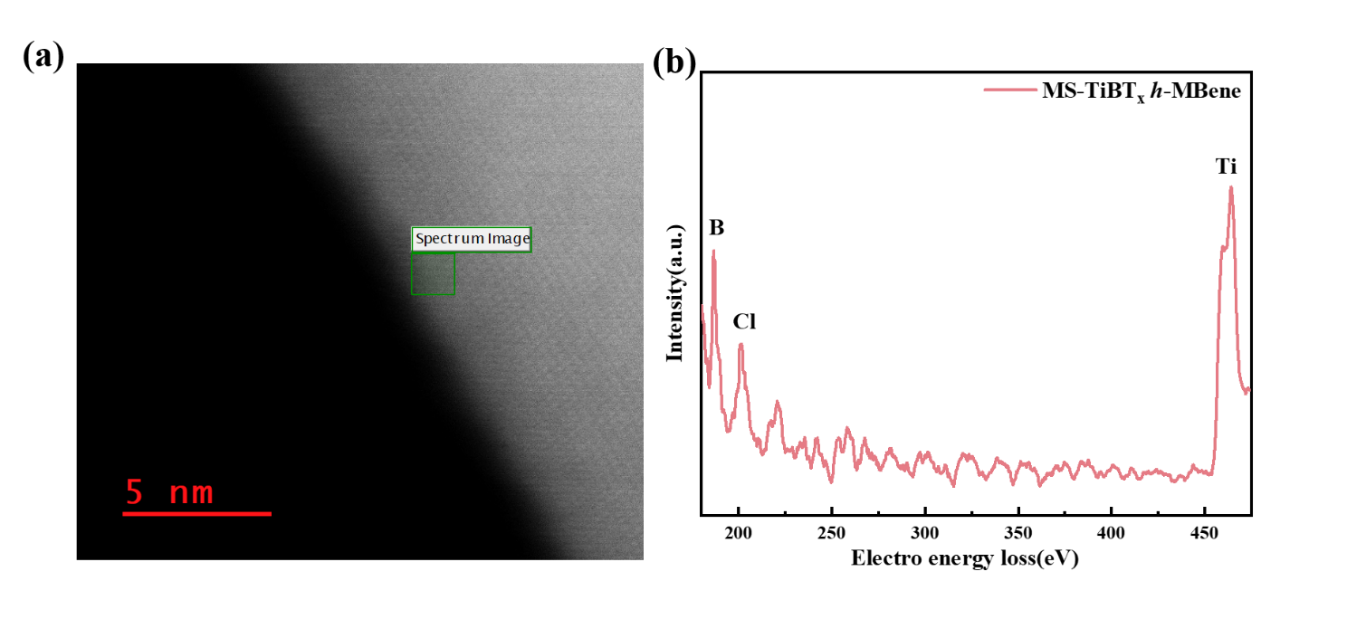


**Figure S8:** (a) STEM image of MS-TiBT_x_ *h*-MBene showing the location of the point scan; (b) EELS spectrum from a MS-TiBT_x_ *h*-MBene point scan.


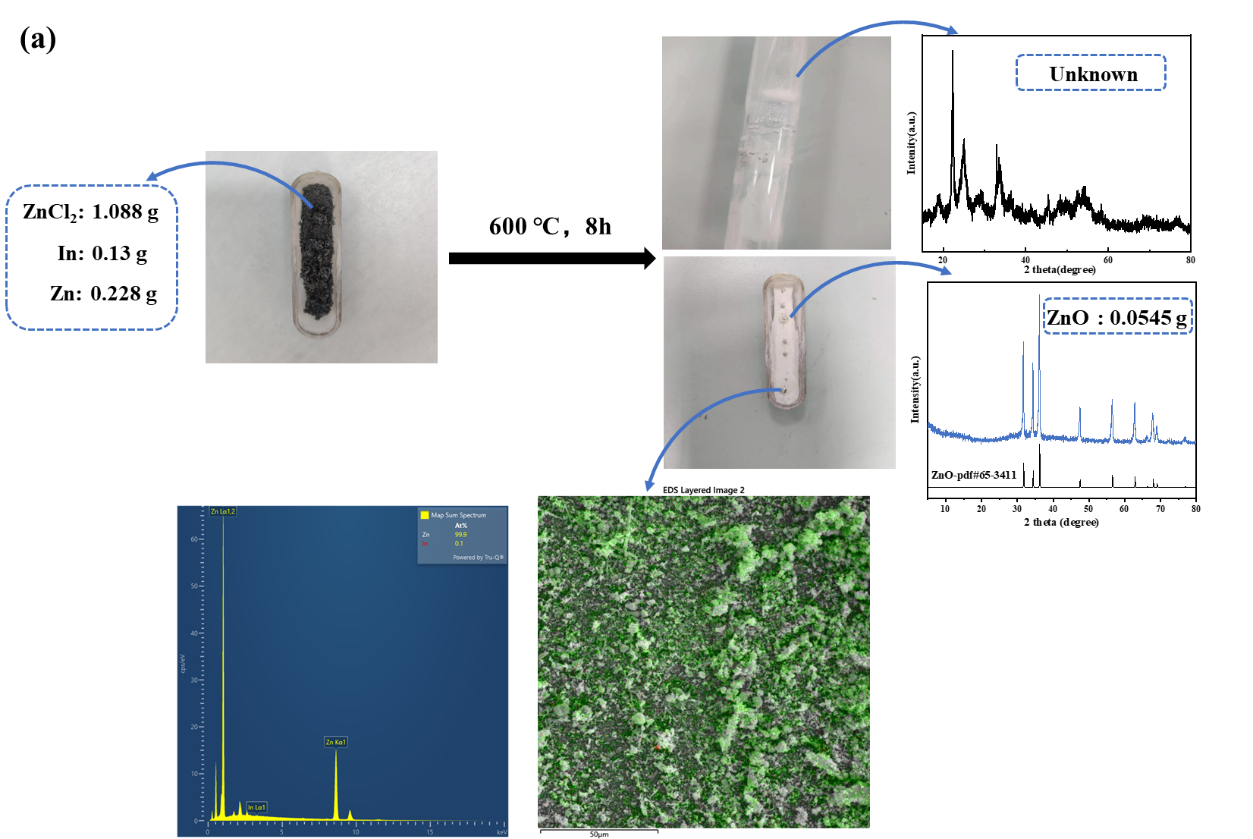


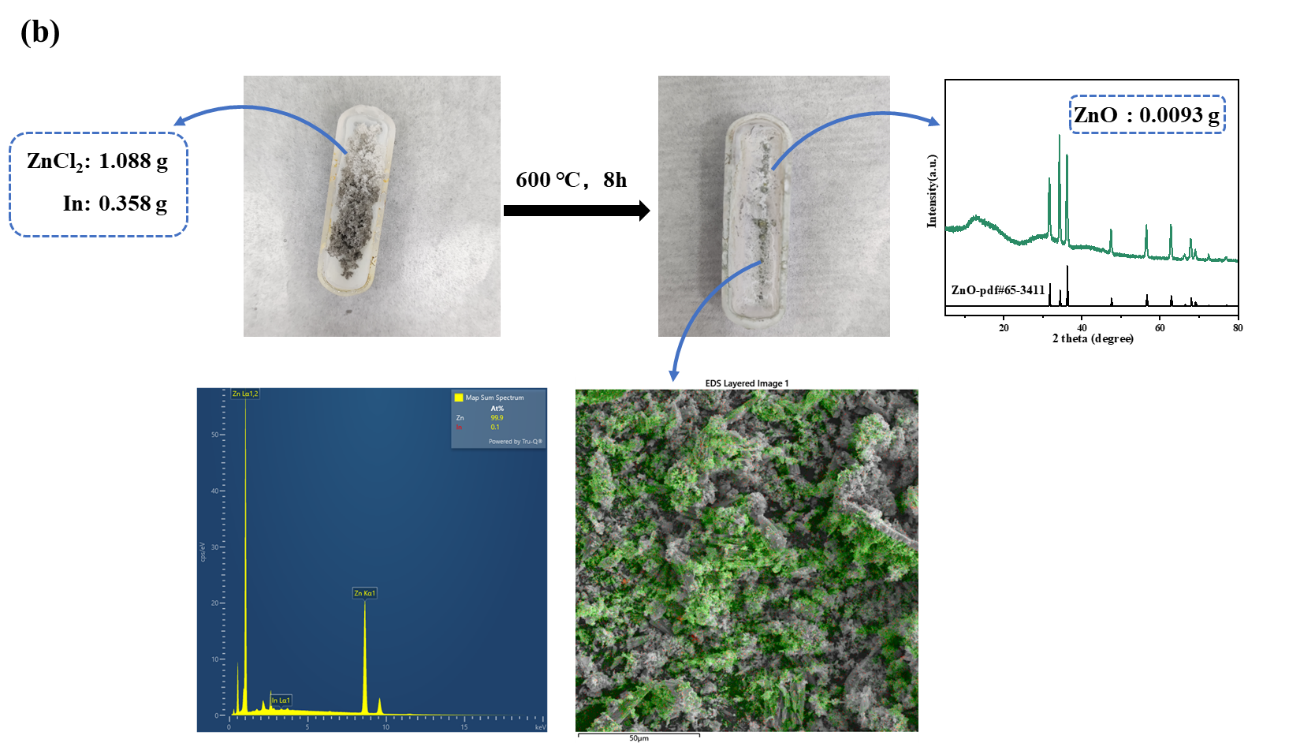


**Figure S9:** Schematic the exploring reaction of (a) ZnIn and ZnCl_2_ and (b) In and ZnCl_2_.

First, ZnCl_2_ (1.088 g), In (0.13 g) and Zn (0.228 g) (1:1:4 molar ratio) elemental powders were mixed and putted into the quartz glass boat. Second, the quartz glass boat with mixed powder heated at 600 ℃ for 8 h. After the reaction, the obtained product was white color, different from dark grey of starting materials, weighted only 0.0545 g. The XRD spectra indicates that the white color species in the quartz glass boat is ZnO.

Additionally, an indeterminate of white material is observed on the walls. To further elucidate the interaction between In and ZnCl_2_, a reaction was conducted under consistent conditions using a mixture of In and ZnCl_2_. The resultant product exhibited a notable decrease in quantity, and XRD analysis confirmed its identity as ZnO. Trace amounts of ZnO were detected, likely attributed to an excess of the reactant ZnCl_2_, which decomposes at elevated temperatures.


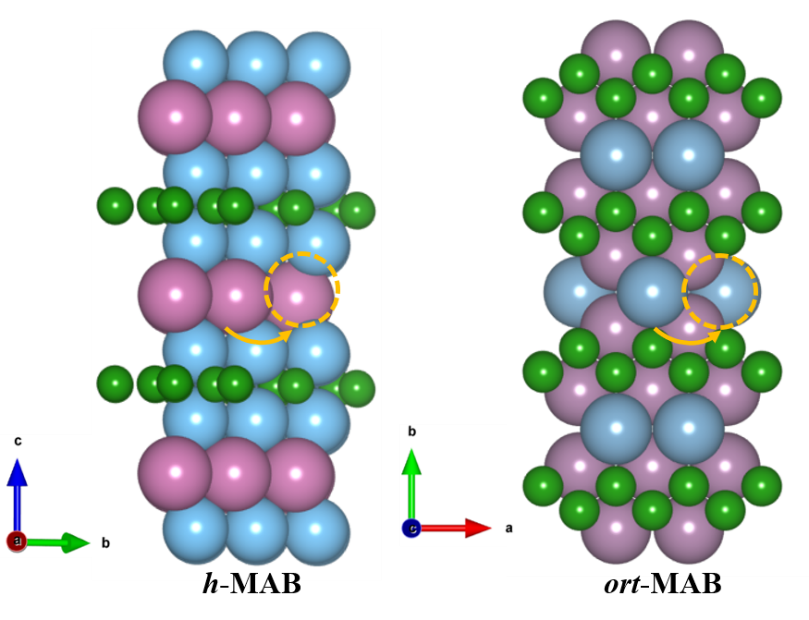


**Figure S10:** Configuration of an A atom (In or Al) migrating along with the A layer of *h*-MAB (Ti_2_InB_2_) or *ort*-MAB (Mo_2_AlB_2_).


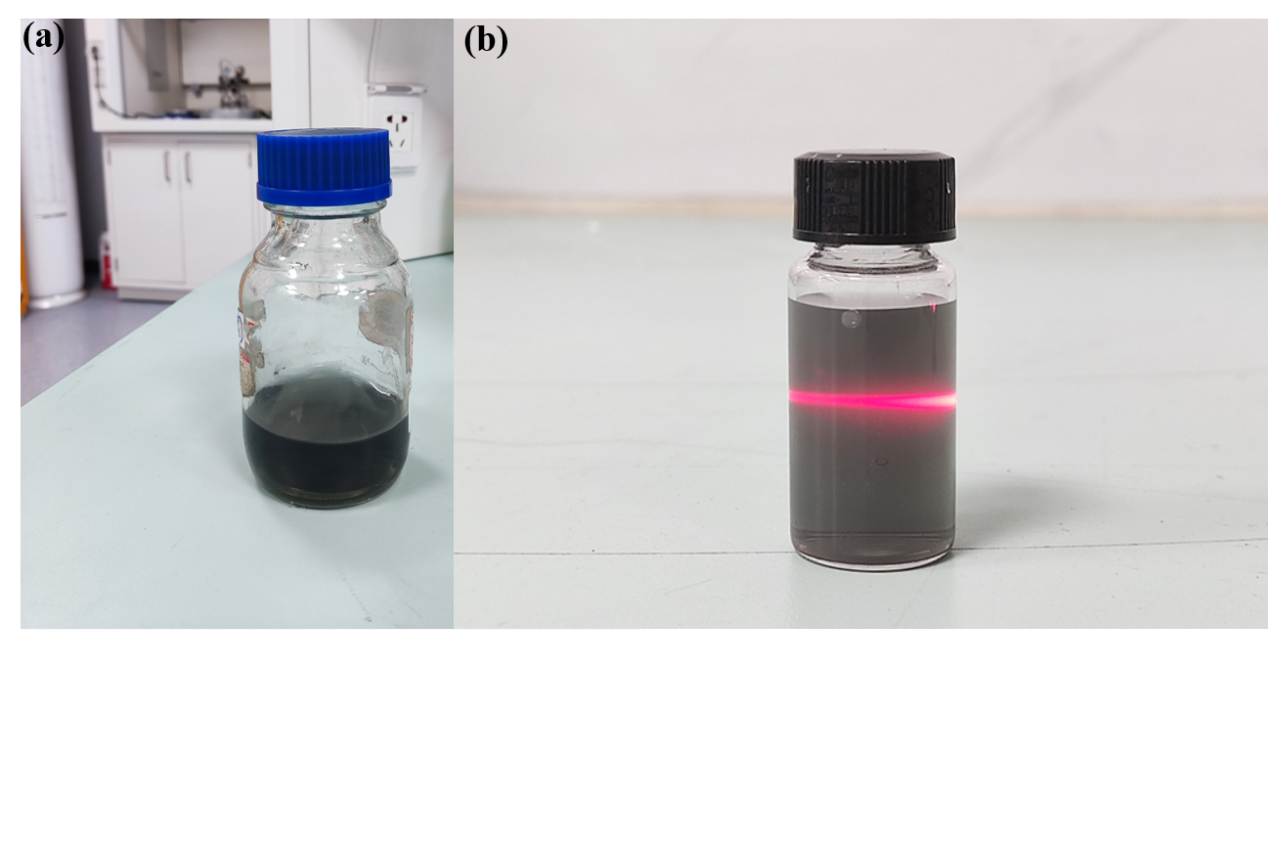


**Figure S11:** (a) Pristine d-TiBT_x_ *h*-MBene after TBAOH treatment and centrifugation at 3500 rpm for 20 min; (b)Tyndall effect for d-TiBT_x_ *h*-MBene nanoflakes dispersed in water after dilution.


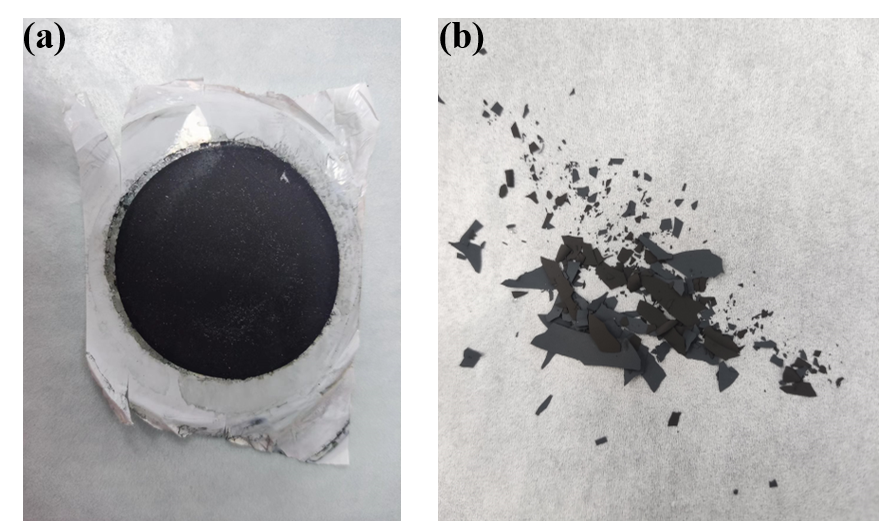


**Figure S12:** (a) The delaminated MS-TiBT_x_ *h*-MBene nanoflakes collection by filtration and (b) after dried in a vacuum oven at 50 ℃ overnight.


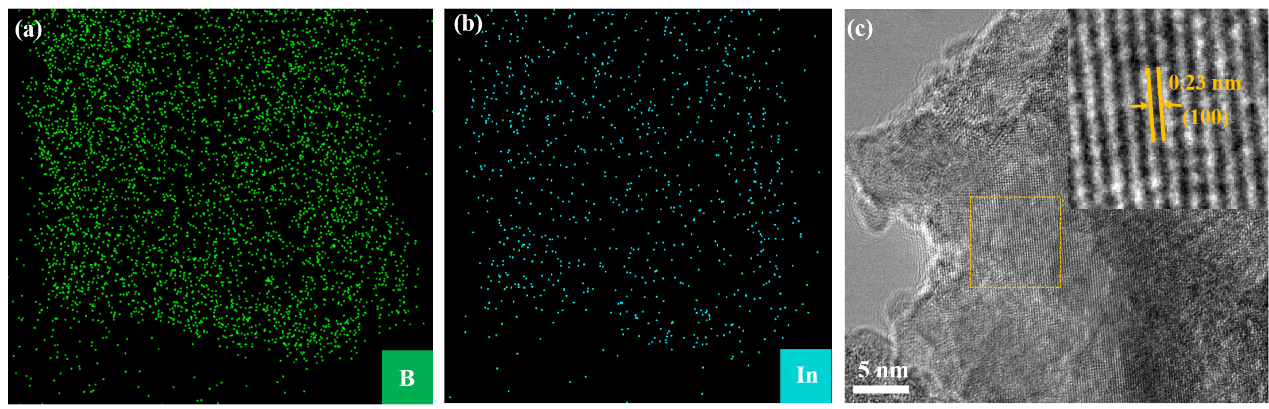


**Figure S13:** (a-b) EDS elemental mapping images of d-TiBT_x_ *h*-MBene flakes.


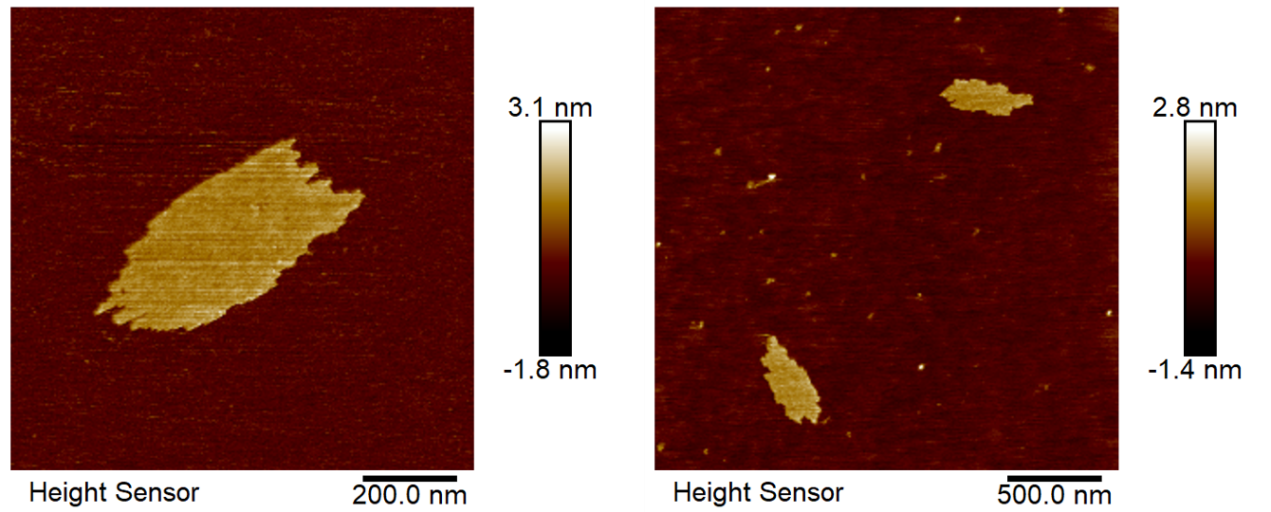


**Figure S14:** AFM images of d-TiBT_x_ *h*-MBene flakes.


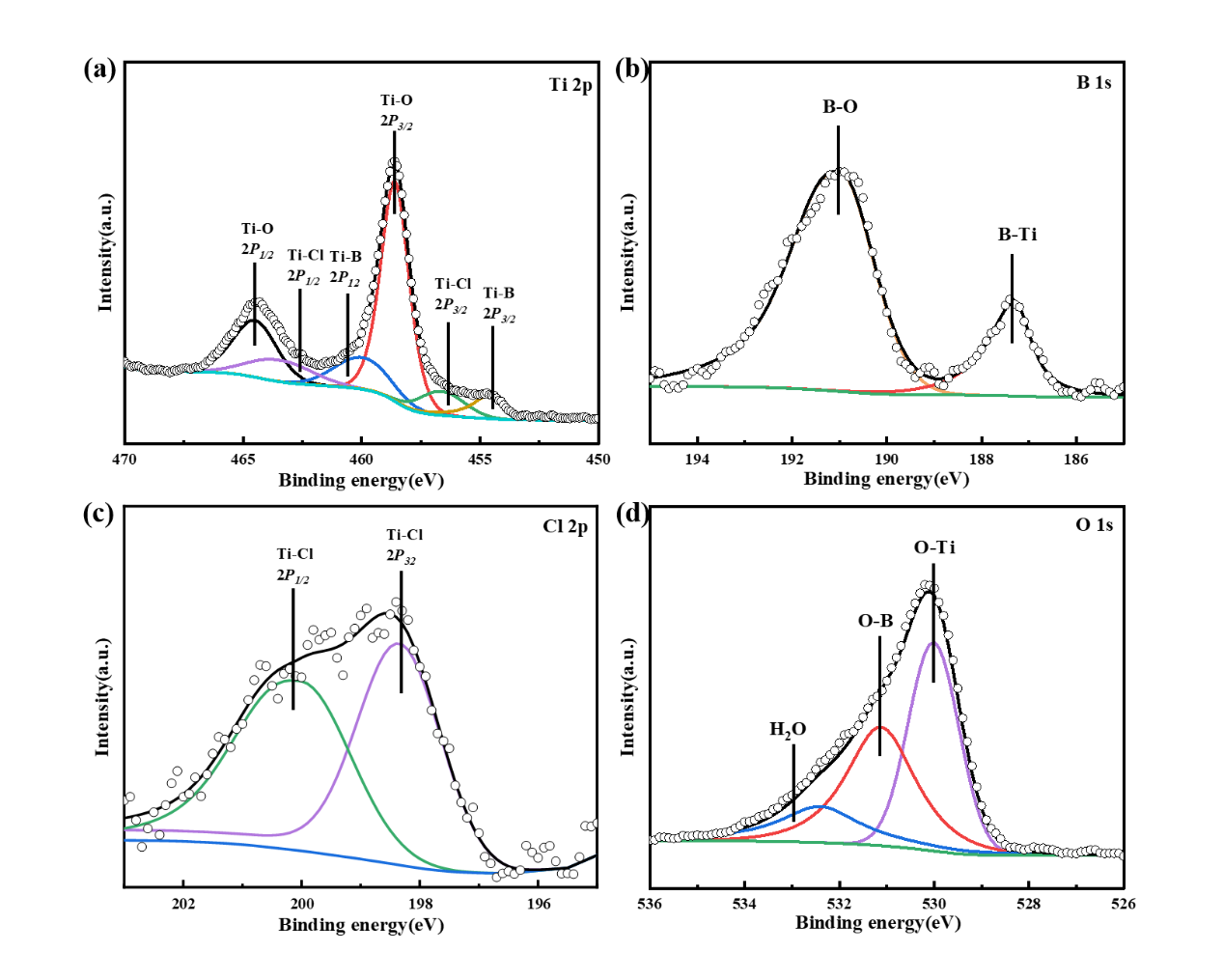


**Figure S15:** (a) XPS survey spectrum and high-resolution XPS spectra of delaminated TiBT_x_ *h*-MBene (a)Ti 2p; (b) B 1s; (c) Cl 2p and (d) O 1s.


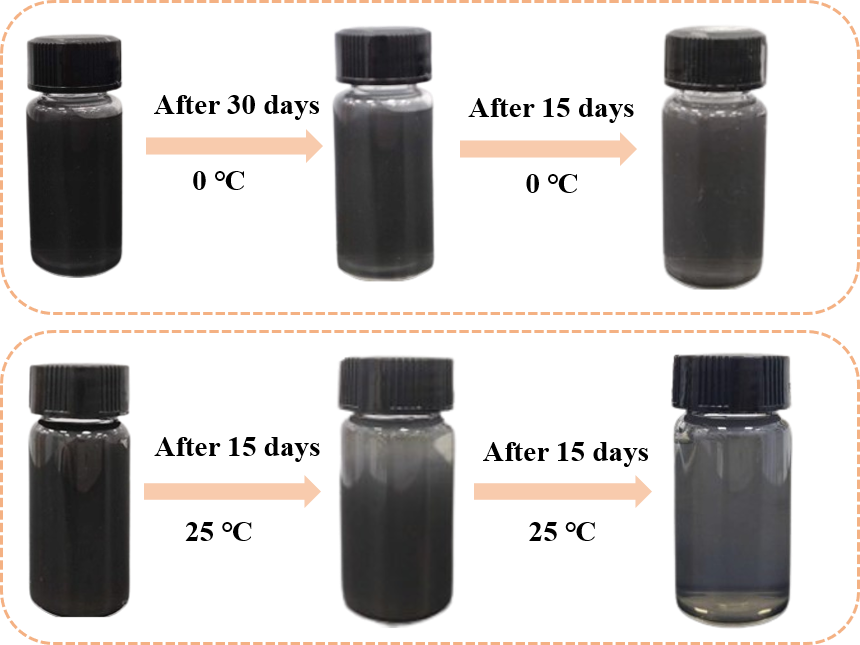


**Figure S16:** Photographs of delaminated TiBT_x_ *h*-MBene water dispersions at the initial state and after 30 days at 0 or 25 ℃.

**Figure S17:** Mean size of d-TiBT_x_ *h*-MBene flakes in water.


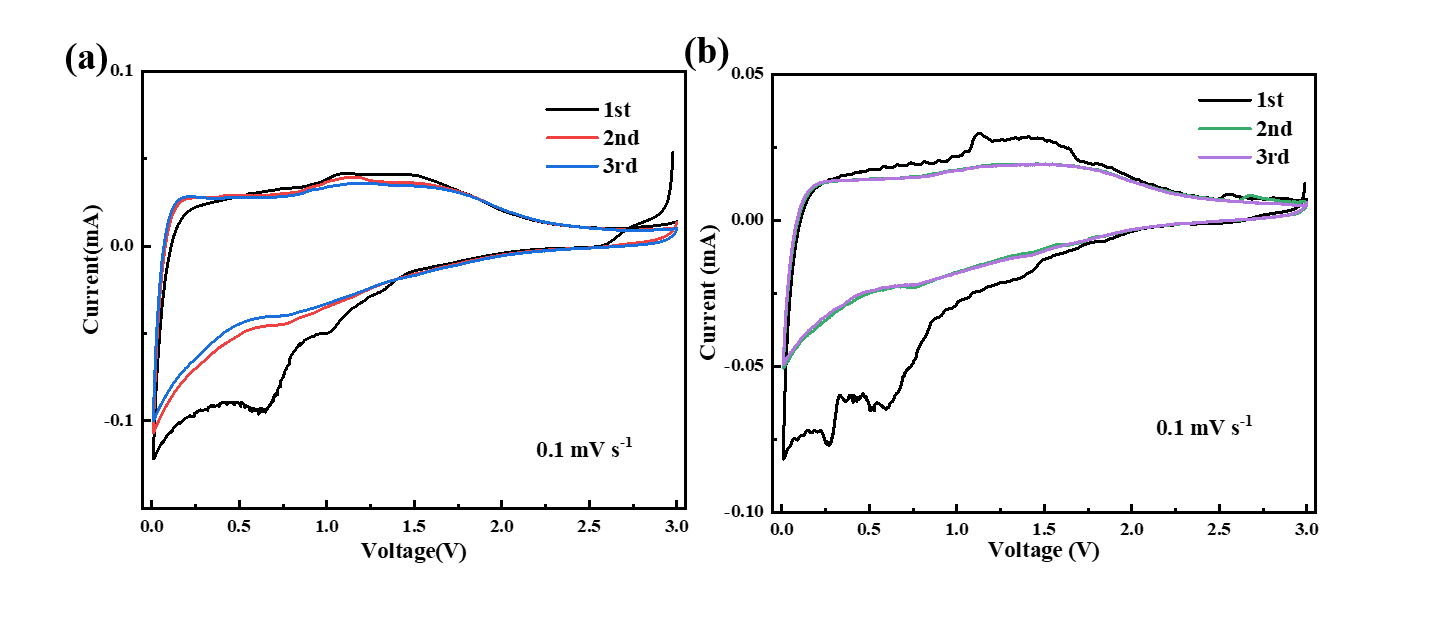


**Figure S18:** (a) First three CV curves of d-TiBT_x_ *h*-MBene electrode and (b) MS-TiBT_x_ *h*-MBene electrode in LIBs at 0.1 mV s^-1^, measured at a scan rate of 0.1 mV s^-1^ within the electrochemical potentials range from 0.01 to 3.0 V vs.Li^+^/Li.

**Figure S19:** Cycling performance of d-TiBT_x_ electrode at current densities of 0.1 A g^−1^.

**Figure S20:** GCD profiles of MS-TiBT_x_ *h*-MBene.

**
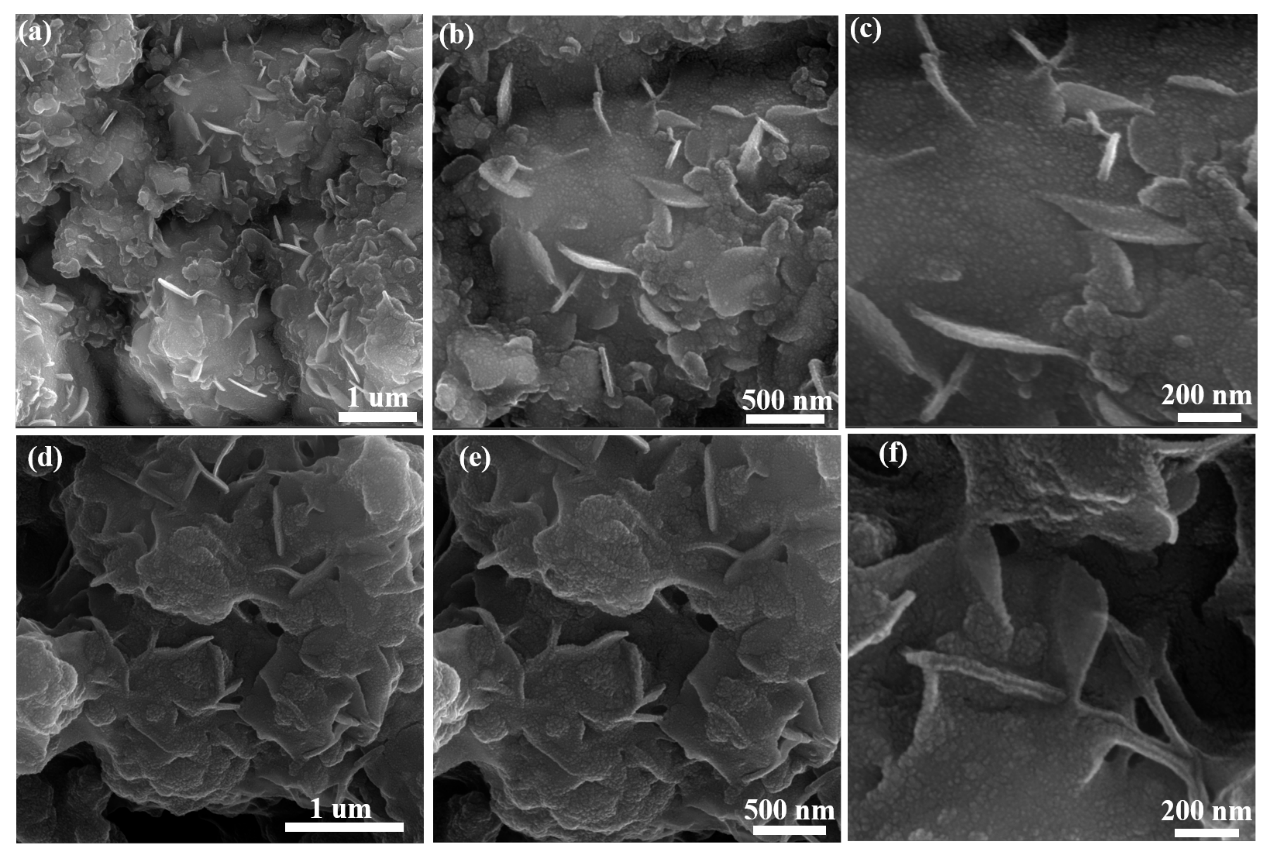
**

**Figure S21**: (a-c) SEM images of d-TiBT_x_ *h*-MBene electrode tested at 1.0 A g^−1^ after 1000 cycles in LIBs.

**
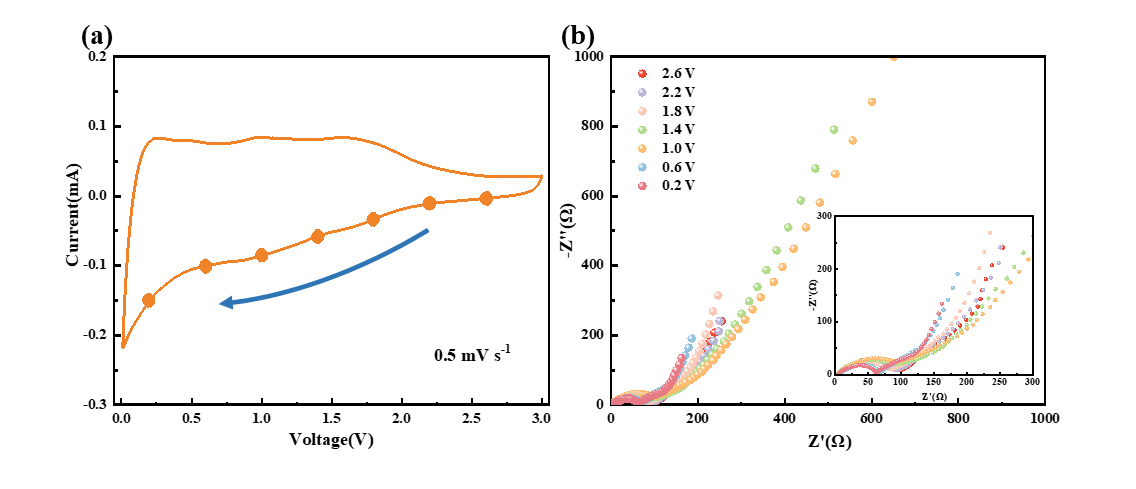
**

**Figure S22:** (a) CV profile recorded at a rate of 0.5 mV s^-1^ and (b)the electrochemical impedance spectroscopy (EIS) plots of d-TiBT_x_ *h*-MBene electrode recorded at various bias potentials.


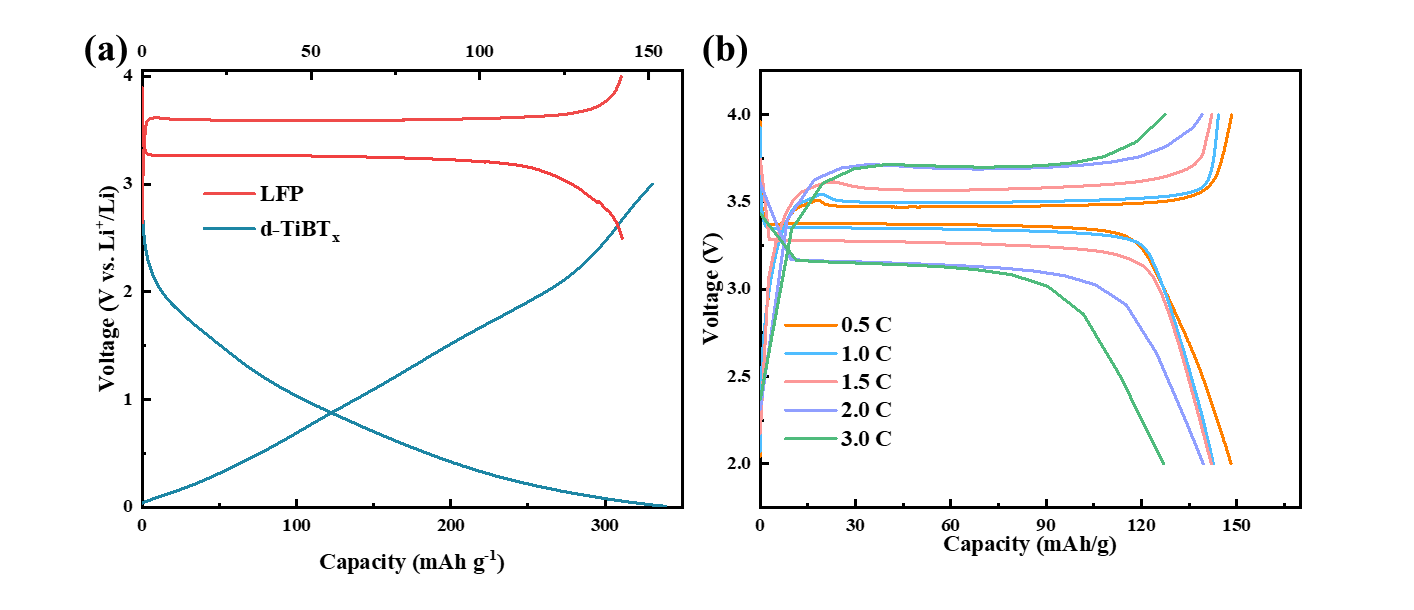


**Figure S23:** (a) Galvanostatic charge/discharge profiles of the d-TiBT_x_ anode and LFP cathode in half-cells. (b) Charge/discharge curves of d-TiBT_x_||LFP LFP full-cell at different current densities.


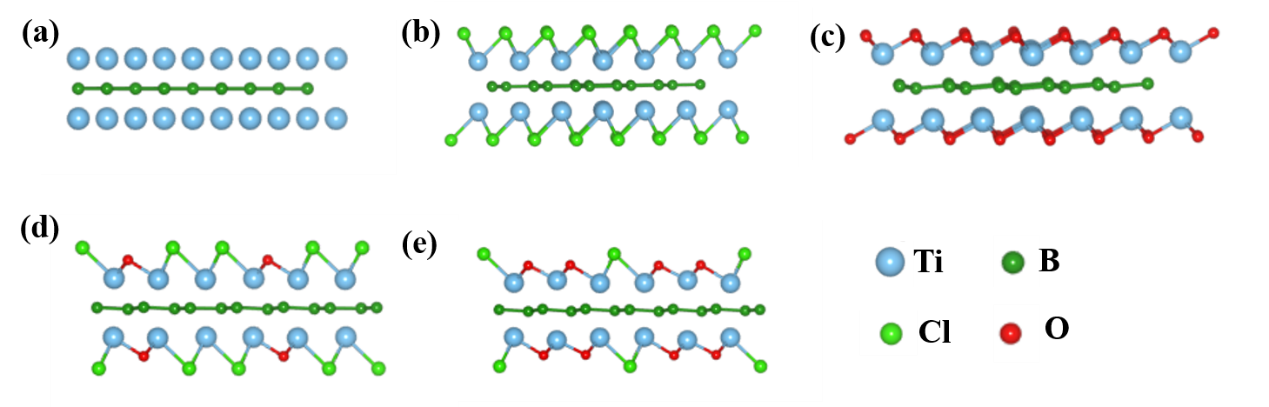


**Figure S24:** The optimized crystal structures of (a) TiB, (b) TiBCl, (c) TiBO, (d) TiBCl_2/3_O_1/3_ and (e) TiBCl_1/3_O_2/3_.


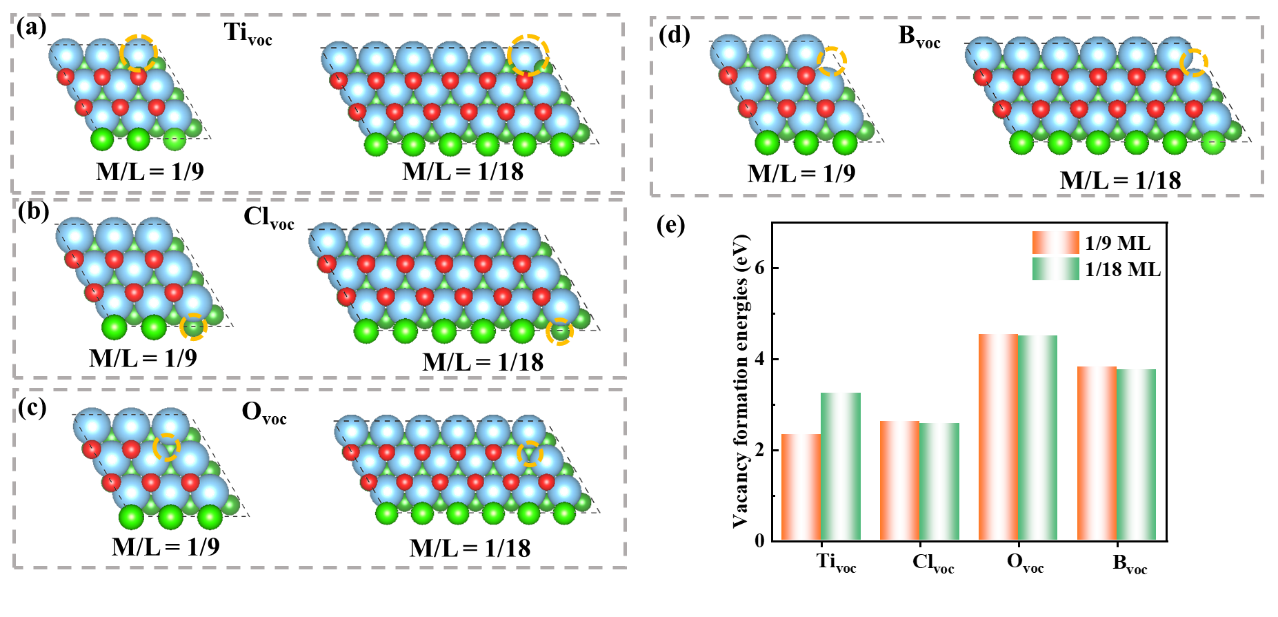


**Figure S25:** Optimized structures of (a) Ti vacancy (Ti_voc_), (b) Cl vacancy (Cl_voc_), (c) O vacancy (O_voc_) and (d) B vacancy (B_voc_). (e) Calculated vacancy formation energies of Ti_voc_, Cl_voc_ ,O_voc_ and B_voc_ with various vacancies coverage.


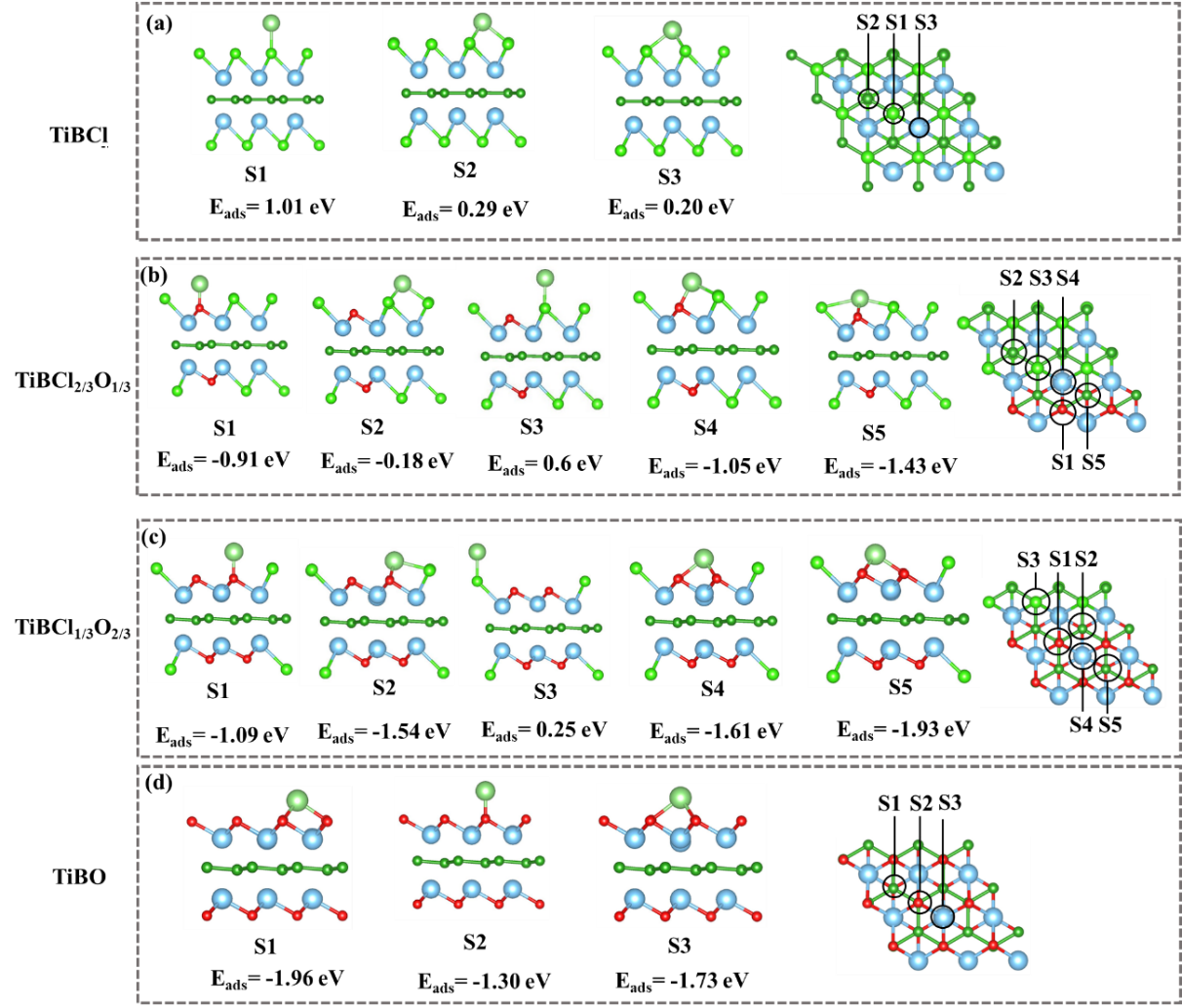


**Figure S26:** The possible adsorption sites and energy of Li on the surface of (a) TiBCl; (b) TiBCl_1/3_O_2/3_;(c) TiBCl_1/3_O_2/3_ and (d) TiBO.

DFT calculations to optimize the most favorable adsorption sites for lithium on the surface of TiBT_x_ with different oxygen-containing functional groups, as shown in **Figure S26**. The results reveal that in TiBCl, the adsorption energy for lithium is positive at all sites, indicating weak stability to adsorp lithium. In contrast, TiBCl_2/3_O_1/3_ and TiBCl_1/3_O_2/3_ exhibit improved adsorption stability when lithium is adjacent to oxygen sites, while adsorption near chlorine sites remains weaker. Notably, TiBO demonstrates negative adsorption energies at all sites, signifying the strongest and most stable lithium adsorption. These findings highlight the high lithiophilic nature of the oxygen-functionalized MBene, where the increased oxygen functional groups significantly enhance lithium-ion interaction. This enhanced interaction is critical for improving both the electrochemical performance and the stability of the solid electrolyte interphase (SEI).


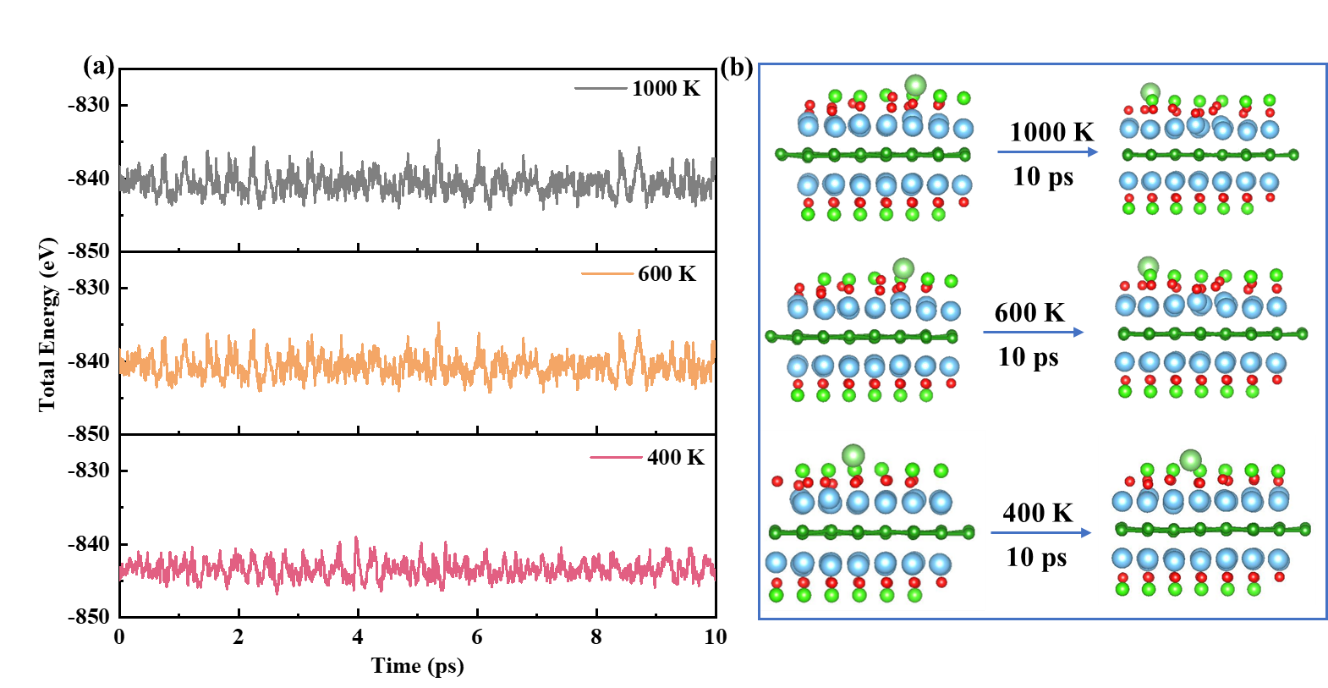


Figure S27: (a) Energy variation of Li-adsorbed TiBCl_1/3_O_2/3_ during AIMD simulations at 500 K, 1000 K, and 1500 K over the time scale of 10 ps; (b) Snapshots of Li-adsorbed TiBCl_1/3_O_2/3_ structure from AIMD simulations at 0 and 10.0 ps at 500 K, 1000 K, and 1500 K.

**Figure S28:** The diffusion paths for Li on the (a)TiBCl_2/3_O_1/3_, (b)TiBCl_1/3_O_2/3_ and(c) TiBO. The green and yellow spheres represent Li atoms.


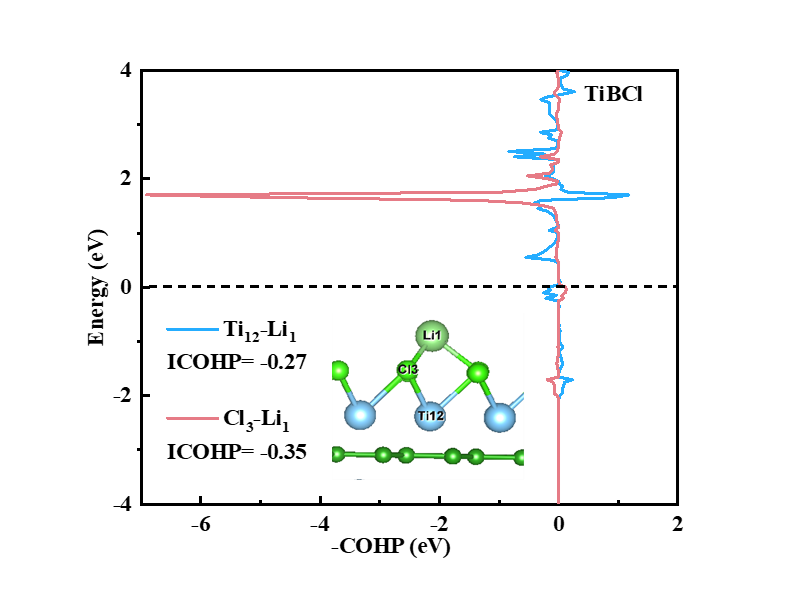


**Figure S29:** COHP between Ti/Cl and Li atoms of Li after adsorption on TiBCl.


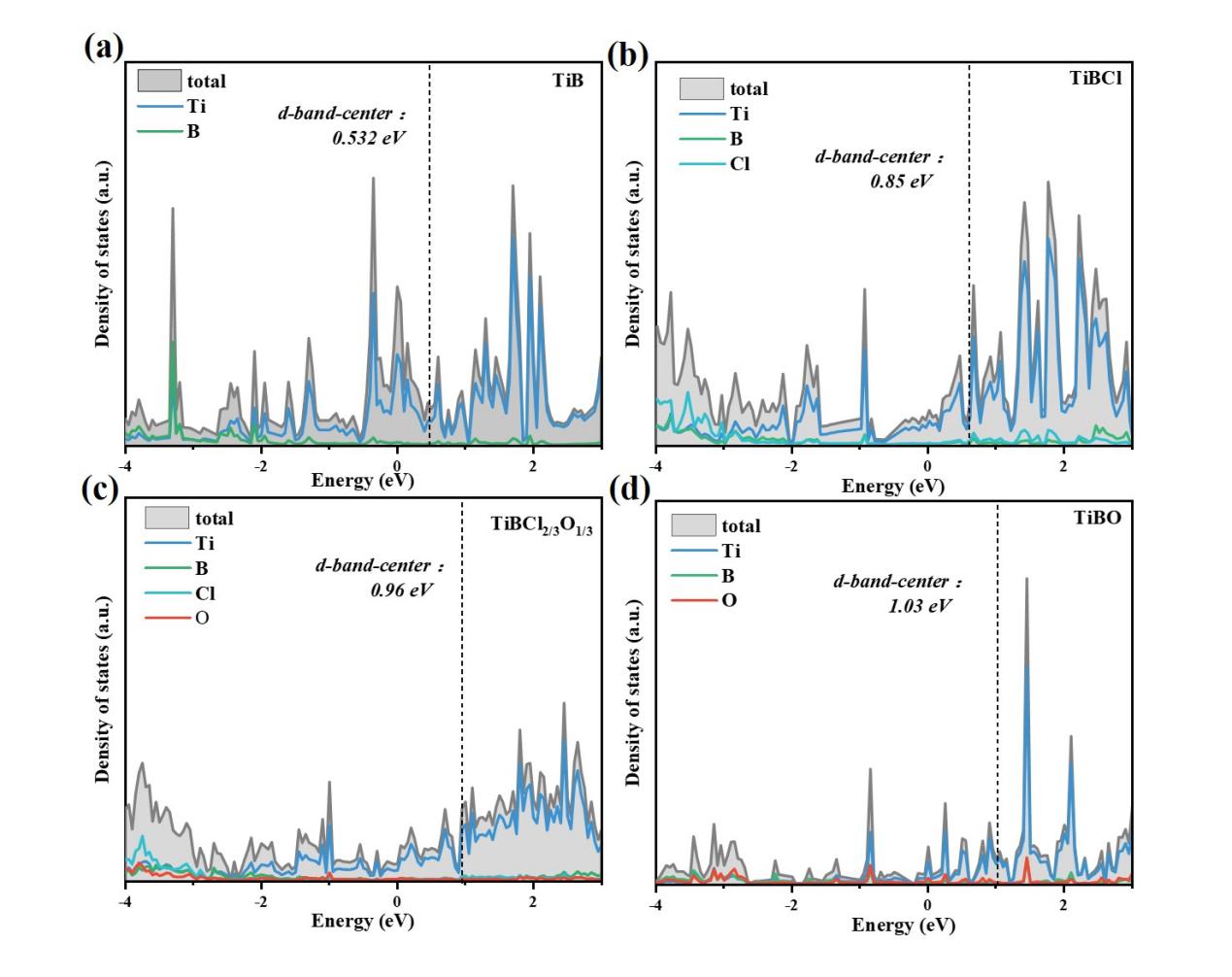


**Figure S30:** The calculated total DOS of (a) TiB, (b) TiBCl, (c) TiBCl_2/3_O_1/3_and (d) TiBO.


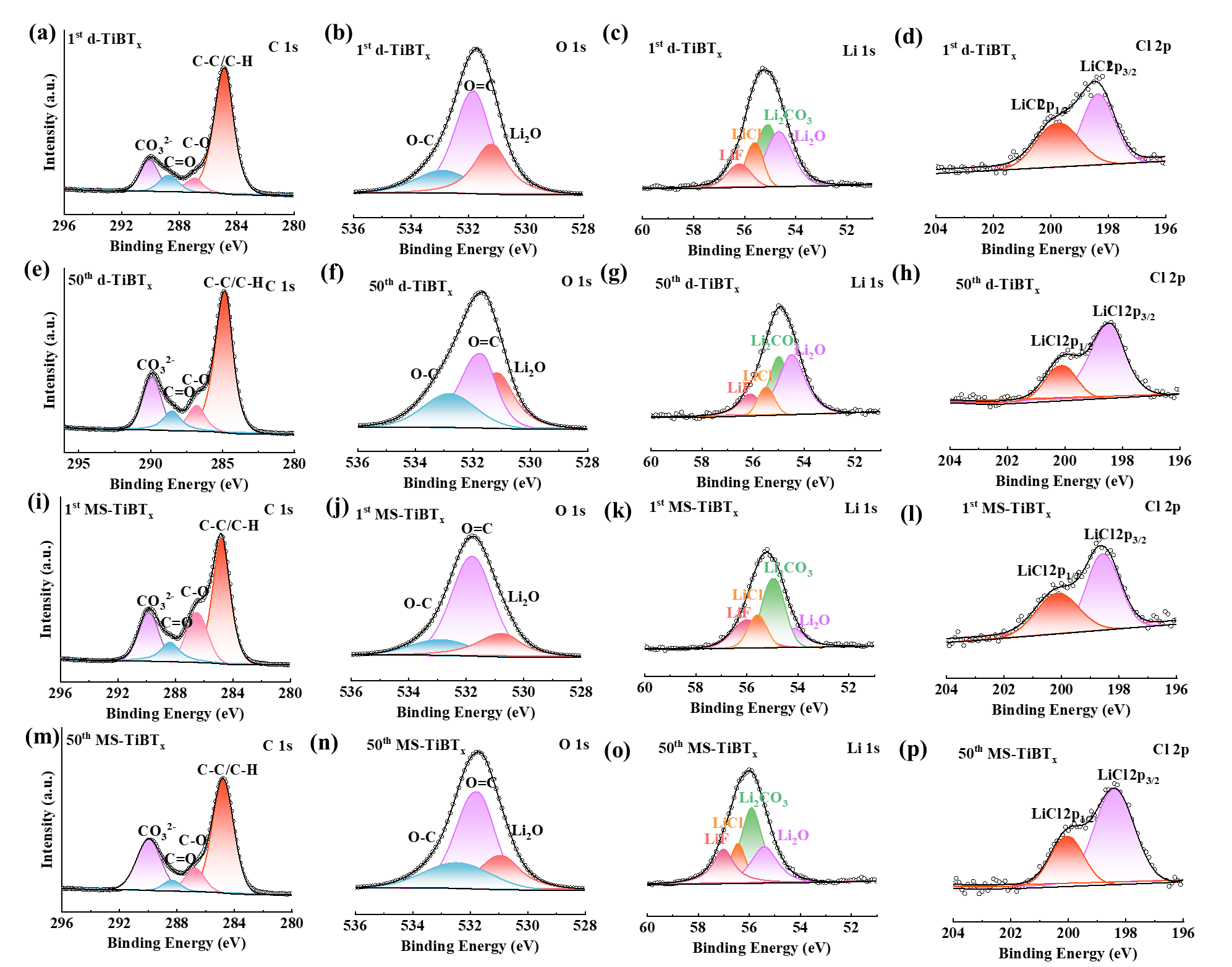


**Figure S31:** XPS spectra of a,e) C 1s, b,f) O 1s, c,g) Li 1s and d,h) Cl 2p taken from cycled 1^st^ and 50 cycles of d-TiBT_x_; XPS spectra of i,m) C 1s, j,n) O 1s, k,o) Li 1s and l,p) Cl 2p taken from cycled 1^st^ and 50 cycles of MS-TiBT_x_.

XPS measurements on the d-TiBTₓ (Ti_1.22_B_1.08_Cl_0.27_O_1.31_) and MS-TiBTₓ (Ti_1.22_B_1.08_Cl_1.02_O_0.49_) electrodes after the first cycle and after 50 cycles at 1 A g⁻¹ to analyze the SEI composition. A series of XPS spectra (C 1s, Li 1s, O 1s and Cl 2p) were collected, as shown in **Figure S31**. As illustrated in **Figures S31a, e, i and m**, both d-TiBTₓ and MS-TiBTₓ exhibited four characteristic peaks at binding energies of 284.9, 285.8, 286.8, and 289.8 eV, which were assigned to C–C & C–H, C–O, O–C–O, and CO_3_^2-^, respectively^3,4^. The relative intensity of the CO_3_^2-^ peak was higher in MS-TiBTₓ, indicating a higher content of Li_2_CO_3_ in the SEI layer, suggesting that the MS-TiBTₓ electrode undergoes more electrolyte decomposition during cycling. In the O 1s spectra (**Figures S31b, f, j and n**), three peaks at 533.2, 532.2, and 529.5 eV were assigned to C–O, C=O, and Li_2_O, respectively^5^. The more intense Li_2_O signal in d-TiBTₓ indicates that the increased oxygen content on the TiB surface promoted the formation of Li_2_O. Additionally, as shown in **Figures S31c, g, k and o**, the Li 1s spectra of both d-TiBTₓ and MS-TiBTₓ electrodes revealed the presence of inorganic compounds such as Li_2_O, Li_2_CO_3_, LiF, and LiCl in the SEI^4^. The Li_2_O content in the SEI of d-TiBTₓ was found to be significantly higher than that in MS-TiBTₓ, further supporting the idea that oxygen-functionalized MBene promotes Li_2_O formation. It is well known that an SEI layer rich in Li_2_O can promote Li-ion transport at the electrode/electrolyte interface due to its ionic conductivity^6,7^. Therefore, the Li_2_O-rich SEI formed on the d-TiBTₓ electrode with rapid ion diffusion kinetics significantly enhances the interfacial performance during cycling. Moreover, the high-resolution Cl 2p spectrum in **Figures S31d, h, l and p** show a doublet with peaks at 198.6/200.2 eV, indicating the presence of LiCl in the SEI layer of both d-TiBTₓ and MS-TiBTₓ electrodes^8^.


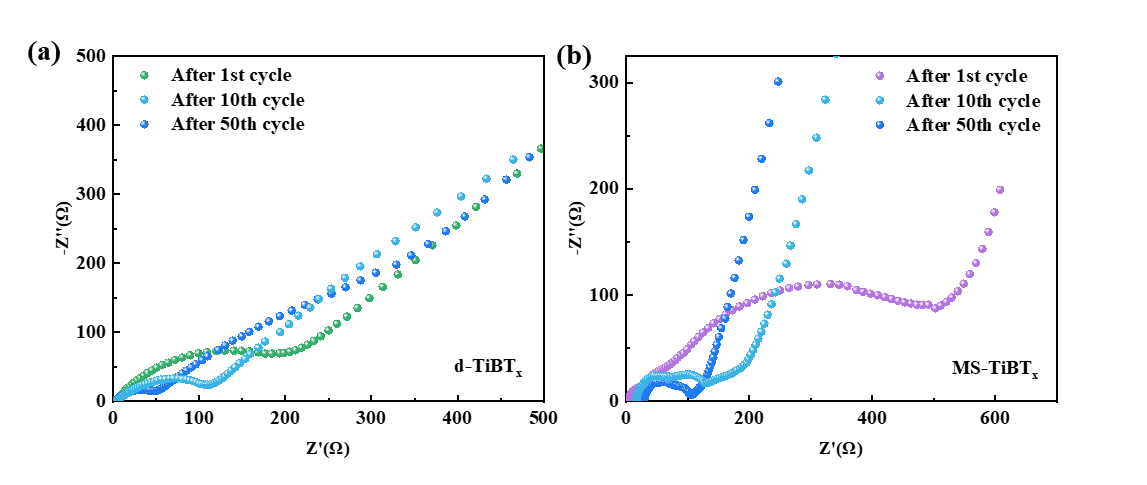


**Figure S32**: EIS measurements of the (a) d-TiBT_x_ and (b) MS-TiBT_x_ electrode after different cycles


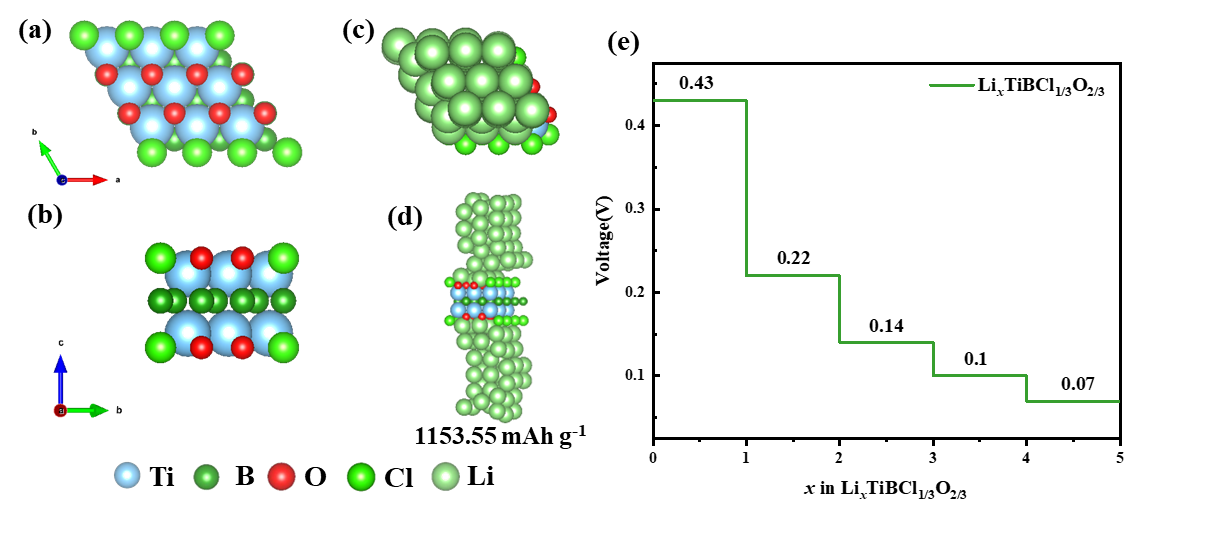


**Figure S33:** (a-b) Optimized structure of TiBCl_1/3_O_2/3_; (c-d) The theoretical Li^+^ capacity of TiBCl_1/3_O_2/3_ monolayers; (e) The theoretical Li^+^ capacity of TiBCl_1/3_O_2/3_ monolayers; and (b) the OCV of the TiBCl_1/3_O_2/3_ monolayer at different Li concentrations.

**Supplementary Table**

**Table S1.** Composition of synthesized Ti_2_InB_2_ measured by XPS.

|  | Ti at% | In at% | | B at% | | O at% | | C at% |
| --- | --- | --- | --- | --- | --- | --- | --- | --- |
| XPS | 27.48 | | 11.38 | | 23.74 | | 4.9 | 32.5 |

**Table S2.** Measured compositions of the MS-TiBT_x_ *h*-MBene

|  | C at% | Ti at% | In at% | B at% | Cl at% | O at% | Zn at% |
| --- | --- | --- | --- | --- | --- | --- | --- |
| XPS | 59.08 | 7.73 | 0.58 | 6.95 | 5.68 | 19.59 | 0.39 |
| EDS | */* | 75.7 | 0.7 | */* | 17.4 | 6.1 | / |
| ICP | */* | 32.435 | 1.195 | 35.185 | / | / | 0.93 |
| EELS | */* | 40.1 | */* | 38.8 | 21.1 | / | / |

The higher C1s peak in MS-TiBT_x_ compared to Ti_2_InB_2_ can be attributed to the increased surface area resulting from the ZnCl_2_ molten salt etching process. This increased surface area makes MS-TiBT_x_ more susceptible to surface contamination from ambient exposure during sample preparation, transfer, and XPS analysis.

**Table S3.** Composition of MS-TiBT_x_ *h*-MBene flakes through measured by EDS and XPS.

|  | | Ti | B | Cl | O | In | | Composition |
| --- | --- | --- | --- | --- | --- | --- | --- | --- |
| EDS | wt./% | 81.91 | / | 13.95 | 2.22 | 1.92 |  | |
|  | mol/% | 1.71 | / | 0.39 | 0.14 | / |  | |
| XPS | wt./% | 44.32 | 11.88 | 35.92 | 7.88 |  | | |
|  | mol/% | 0.94 | 1.08 | 1.02 | 0.49 | **Ti_1.22_B_1.08_Cl_1.02_O_0.49_** | | |

**Table S4.** Gibbs free energy (Δ*G*, kJ mol^-1^) of the reaction of different Lewis molten salts (ZnCl_2_, CuCl_2_, MnCl_2_, FeCl_2_ and NiCl_2_) with the In atoms of Ti_2_InB_2_ at 600°C.

|  | ZnCl_2_ | CuCl_2_ | | FeCl_2_ | | CoCl_2_ | | MnCl_2_ |
| --- | --- | --- | --- | --- | --- | --- | --- | --- |
| In | -388.149 | | -206.65781 | | -324.78605 | | 296.78 | 186.14405 |

**Table S5**. Comparison of the electrochemical performances of d TiBT_x_ *h*-MBene anode with those reported molten salt-derived MXenes anode for LIBs.

| **Materials** | **Max Capacity**  **(mAh g^-1^)** | **Rate capacity**  **(mAh g^-1^)** | **Cycling Performance** | | | | | **Reference** |  |
| --- | --- | --- | --- | --- | --- | --- | --- | --- | --- |
|  |  |  | **Rate** | **Cycle Number** | | **Capacity**  **(mAh g^-1^) (retention%)** | |  |  |
| OBO- Ti_3_C_2_ | 300 (0.1 A/g) | 202 (1 A/g) | 1 A/g | | 2000 | | 180  (94%) | *Nat. Mater (****2024****)*^9^ |  |
| Ti_2_CCl_2_ MXenes. | | 286 (0.1 A/g) | 70 (10 A/g) | / | | / | | / | *Science (****2023****)*^10^ |
| Ti_3_C_2_ MXene | 207 (0.1 A/g) | 125 (1 A/g) | 3 A/g | | 2400 | | 90  (90%) | *Nat. Mater (****2020****)*^11^ |  |
| Ti_2_C MXene | 280 (0.1 A/g) | 80 (10 A/g) | / | | / | | / | *Nat. Commun (****2021****)*^12^ |  |
| HfBO  *h*-MBene | 420 (0.1 A/g) | 125 (1 A/g) | / | | / | | / | *Angew. Chem. Int. Ed (****2023****)^13^* |  |
| e-MS-Ti_3_C_2_T_x_ | 225 (0.2 A/g) | 105 (8 A/g) | 4 A/g | | 2000 | | 85  (72%) | *ACS Nano(****2022****)* ^14^ |  |
| Ti_3_C_2_Cl_x_ MXene | 180 (0.1 A/g) | 85 (2 A/g) | 1 A/g | | 500 | | 115  (95.8%) | *Electrochemisty Communicatios (****2022****)*^15^ |  |
| Nb_2_CT_x_ MXene | 330 (0.05A/g) | 80 (10 A/g) | 1 A/g | | 1600 | | 190  (130%) | *ChemElectroChem (****2021****)*^16^ |  |
| d-TiBT_x_  *h*-MBene | 530 (0.1 A/g) | 120 (10 A/g) | 1 A/g | | **1000** | | **254**  **(97.7%)** | **This work** |  |

**Table S6.** Average chemical compositions (at%) of Ti_2_InB_2_ and MS-TiBT_x_ *h*-MBene and delaminated TiBT_x_ *h*-MBene flakes through measured by EDS.

| Samples | Ti | In | Cl | O |
| --- | --- | --- | --- | --- |
| Ti_2_InB_2_ | 66.8 | 33.2 | / | / |
| MS-TiBT_x_ *h*-MBene | 75.7 | 0.7 | 17.4 | 6.1 |
| d-TiBT_x_ *h*-MBene | 53.2 | 0.2 | 3.7 | 42.9 |

**Table S7.** Composition of delaminated TiBT_x_ *h*-MBene flakes through measured by EDS and XPS.

|  | | Ti | B | Cl | O | Composition |
| --- | --- | --- | --- | --- | --- | --- |
| EDS | wt./% | 75.23 | / | 3.84 | 20.26 |  |
|  | mol/% | 1.6 | / | 0.11 | 1.2 |  |
| XPS | wt./% | 57.6 | 11.72 | 9.61 | 21.07 |  |
|  | mol/% | 1.22 | 1.08 | 0.27 | 1.31 | **Ti_1.22_B_1.08_Cl_0.27_O_1.31_** |

**References**

1. Zhou, J. *et al.* Boridene: two-dimensional Mo_4/3_B_2-x_ with ordered metal vacancies obtained by chemical exfoliation. *Science* **373**, 801–805 (2021).

2. Dahlqvist, M. *et al.* Out-Of-Plane ordered laminate borides and their 2D Ti-Based derivative from chemical exfoliation. *Adv. Mater* **33**, 2008361 (2021).

3. Oyakhire, S. T., Gong, H., Cui, Y., Bao, Z. & Bent, S. F. An X-ray Photoelectron Spectroscopy Primer for Solid Electrolyte Interphase Characterization in Lithium Metal Anodes. *ACS Energy Lett.* **7**, 2540–2546 (2022).

4. Jin, Q. *et al.* Tuning solvation behavior within electric double layer via halogenated MXene for reliable lithium metal batteries. *Energy Storage Mater.*  **73**, 103837 (2024).

5. Hui, X. *et al.* Oxide Nanoclusters on Ti_3_ C_2_ MXenes to Deactivate Defects for Enhanced Lithium Ion Storage Performance. *Small* **18**, 2104439 (2022).

6. Wu, S. *et al.* A Multifunctional Molecular Modulated Strategy Featuring Novel Li^+^ Transport Centers and Li_2_ O‐Rich SEI Layer for High‐Performance All‐Solid‐State Lithium Metal Batteries. *Angew. Chem. Int. Ed.* e202422942 (2025)

7. Hao, Z., Liu, D., Zuo, X., Yu, H. & Zhang, Y. Unveiling the In Situ Evolution of Li_2_ O‐Rich Solid Electrolyte Interface on CoO*_x_* Embedded Carbon Fibers as Li Anode Host. *Adv. Mater* 2404983 (2024).

8. Ma, L. *et al.* Enabling Stable and Low-Strain Lithium Plating/Stripping with 2D Layered Transition Metal Carbides by Forming Li-Zipped MXenes and a Li Halide-Rich Solid Electrolyte Interface. *Angew. Chem. Int. Ed.* **63**, e202318721 (2024).

9. Li, D. *et al.* MXenes with ordered triatomic-layer borate polyanion terminations. *Nat. Mater.* **23**, 1085-1092 (2024).

10. Wang, D. *et al.* Direct synthesis and chemical vapor deposition of 2D carbide and nitride MXenes. *Science* **379**, 1242–1247 (2023).

11. Li, Y. *et al.* A general lewis acidic etching route for preparing MXenes with enhanced electrochemical performance in non-aqueous electrolyte. *Nat. Mater.* **19**, 894–899 (2020).

12. Ma, G. *et al.* Li-ion storage properties of two-dimensional titanium-carbide synthesized via fast one-pot method in air atmosphere. *Nat Commun* **12**, 5085 (2021).

13. Miao, N. *et al.* Discovery of two‐dimensional hexagonal MBene HfBO and exploration on its potential for lithium‐ion storage. *Angew. Chem. Int. Ed.* **135**, e202308436 (2023).

14. Liu, L. *et al.* Exfoliation and delamination of Ti_3_C_2_T*_x_* MXene prepared *via* molten salt etching route. *ACS Nano* **16**, 111–118 (2022).

15. Liu, P., Guan, B., Lu, M., Wang, H. & Lin, Z. Influence of aqueous solutions treatment on the Li^+^ storage properties of molten salt derived Ti_3_C_2_Cl*_x_* MXene. *Electrochemistry Communications* **136**, 107236 (2022).

16. Dong, H. *et al.* Molten salt derived Nb_2_CT_x_ MXene anode for Li-ion batteries. *ChemElectroChem* **8**, 957–962 (2021).
